# Prospective evaluation of plasma proteins in relation to surgical endometriosis diagnosis in the Nurses’ Health Study II

**DOI:** 10.1016/j.ebiom.2025.105688

**Published:** 2025-04-11

**Authors:** Naoko Sasamoto, Long H. Ngo, Allison F. Vitonis, Simon T. Dillon, Maryam Aziz, Amy L. Shafrir, Stacey A. Missmer, Towia A. Libermann, Kathryn L. Terry

**Affiliations:** a Department of Obstetrics and Gynecology, Brigham and Women’s Hospital and Harvard Medical School, Boston, MA, USA; b Boston Center for Endometriosis, Boston Children's Hospital and Brigham and Women's Hospital, Boston, MA, USA; c Department of Medicine, Beth Israel Deaconess Medical Center and Harvard Medical School, Boston, MA, USA; d Department of Biostatistics, Harvard T.H. Chan School of Public Health, Boston, MA, USA; e Genomics, Proteomics, Bioinformatics and Systems Biology Center, Beth Israel Deaconess Medical Center, Boston, MA, USA; f Department of Population Health Sciences, Duke University, USA; g Department of Health Sciences and Nutrition, School of Nursing and Health Sciences, Merrimack College, North Andover, MA 01845, USA; h Division of Adolescent and Young Adult Medicine, Boston Children’s Hospital and Harvard Medical School, Boston, MA, USA; i Department of Obstetrics, Gynecology, and Reproductive Biology, Michigan State University, Grand Rapids, MI, USA; j Department of Epidemiology, Harvard T.H. Chan School of Public Health, Boston, MA, USA

**Keywords:** Endometriosis, Proteomics, Biomarker, Prospective cohort, Systemic immune dysregulation

## Abstract

**Background:**

Endometriosis is a chronic inflammatory condition characterised by pain and infertility. We conducted a prospective study to elucidate the pathophysiological mechanisms underlying endometriosis development.

**Methods:**

We examined the association between 1305 proteins measured by SomaScan proteomics and risk of endometriosis diagnosis in prospectively collected plasma from 200 laparoscopically-confirmed endometriosis cases and 200 risk-set sampling matched controls within the Nurses’ Health Study II (NHSII) cohort. Using conditional logistic regression, we calculated odds ratios (OR) and 95% confidence intervals (CI) per one standard deviation increase in protein levels and area under the curve (AUC) to assess the multi-protein model in discriminating cases from controls. Analytical validation for three proteins was performed using immunoassays. Ingenuity Pathway Analysis and STRING analyses identified biological pathways and protein interactions.

**Findings:**

Blood samples from cases were collected up to 9 years before diagnosis (median = 4 years). Among 61 individual proteins nominally significantly associated with risk of endometriosis diagnosis compared to controls, endometriosis cases had higher plasma levels of S100A9 (OR = 1.52, 95%CI = 1.19–1.94), ICAM2 (OR = 1.47, 95%CI = 1.17–1.85), HIST1H3A (OR = 1.42, 95%CI = 1.31–1.78), TOP1 (OR = 1.95, 95%CI = 1.24–3.06), CD5L (OR = 1.23, 95%CI = 1.00–1.51) and lower levels of IGFBP1 (OR = 0.70, 95%CI = 0.52–0.94). We further evaluated three of the proteins in an independent set of 103 matched case–control pairs within the NHSII cohort. Pathway analyses revealed upregulation of multiple immune-related pathways in blood samples collected years before endometriosis diagnosis.

**Interpretation:**

In this prospective analysis using aptamer-based proteomics, we identified multiple proteins and biological pathways related to innate immune response upregulated years before endometriosis surgical diagnosis, suggesting the role of immune dysregulation in endometriosis development.

**Funding:**

This study was supported by the 10.13039/100000005Department of Defence, the 2017 Boston Center for Endometriosis Trainee Award. Investigators were supported by Aspira Women’s Health and 10.13039/100000002NIH which were not directly related to this project.

**Research in context**

**Evidence before this study**

Endometriosis is a painful chronic inflammatory condition whose aetiology is not fully understood. Most prior blood-based biomarker studies in endometriosis used a cross-sectional design which included prevalent cases that were largely diagnosed years after symptom onset rather than earlier in the disease trajectory.

**Added value of this study**

This nested case–control study using prospectively collected blood samples comprehensively investigated the association between plasma proteomic biomarkers and biological pathways dysregulated before endometriosis diagnosis. We found upregulation of multiple immune-related pathways in blood samples collected years before endometriosis diagnosis, suggesting these pathways contribute to development of endometriosis. Our results provide evidence that could lead to risk stratification to enhance earlier diagnosis or potential new therapeutic targets for prevention and earlier intervention.

**Implications of all the available evidence**

This prospective study examining proteomics profiles in blood samples collected up to 9 years prior to endometriosis diagnosis demonstrated proteins related to innate immune response are upregulated years before endometriosis diagnosis. These findings provide evidence supporting immune dysregulation as a key element of endometriosis pathogenesis. Replication of these results are required in independent study populations, as well as mechanistic studies in cell lines and animal models.

## Introduction

Endometriosis is a chronic inflammatory condition, defined by the presence of endometrium-like tissue outside the uterus, that often presents with life-impacting pelvic pain and greater risk of infertility, burdening approximately 200 million women worldwide.^1^ Although menstrual pain during adolescence is a frequent early symptom of endometriosis, the requirement of surgical visualisation is a barrier to timely diagnosis, leading to an average of seven-year delay from symptom onset to diagnosis.^2^^,^^3^ Consequently, women with endometriosis suffer from prolonged pain, decreased quality of life, and increased risk of disease comorbidities, resulting in substantial social and economic burden.^2^^,^^4^^,^^5^ Thus, there is a critical need to identify non-invasive biomarkers for earlier diagnosis of endometriosis. However, most prior studies utilised samples from prevalent endometriosis cases (women already diagnosed with endometriosis), limiting the ability to discover non-invasive biomarkers that may indicate future disease years prior to diagnosis. Discovery of biomarkers associated with endometriosis diagnosis years later would shift the clinical diagnosis earlier in the disease trajectory as is desperately needed.

Furthermore, the aetiology of endometriosis is not fully understood. Retrograde menstruation, which is described as the backflow of menstrual endometrial tissue through the fallopian tubes and results in implants of endometrial tissue fragments and cells on the peritoneal surface, is one explanation of ectopic endometriotic lesion development in endometriosis.^6^^,^^7^ Studies show that up to 90% of women have retrograde menstruation but only ∼10% develop endometriosis. This discrepancy suggests that the peritoneal milieu in women who develop endometriosis are more hospitable to the survival of ectopic endometrial tissues.^8^ Prior studies also report differences in circulating inflammatory biomarkers between those with and without endometriosis.^9^, ^10^, ^11^, ^12^ However, all but one study used a cross-sectional design comparing blood inflammatory biomarkers in prevalent endometriosis cases vs. controls, limiting the ability to disentangle the temporality of the association; whether the observed differences in blood biomarkers are risk biomarkers for endometriosis or if they are consequences of having endometriosis disease for multiple years since symptom onset. Here, we conducted a prospective study to comprehensively examine and identify plasma proteomic profiles in blood samples collected years prior to surgical diagnosis of endometriosis to identify candidate blood-based biomarkers for earlier diagnosis of endometriosis and provide insight in the pathophysiology of endometriosis disease development.

## Methods

### Study population

The Nurses’ Health Study II (NHSII) is a USA-based prospective cohort study established in 1989, that enrolled 116,429 registered nurses aged 25–42 years from 14 states.^13^ The participants have been followed by biennial questionnaires assessing updated exposure information on lifestyle factors and ascertaining disease diagnosis, with a high response rate of >90%. Physician-diagnosed endometriosis was first assessed by self-report in 1993 and thereafter in the biennial questionnaires. If a participant reported they had physician-diagnosed endometriosis, they reported the year of diagnosis and whether it was confirmed by laparoscopy, as this is the current clinical standard for endometriosis diagnosis.^14^, ^15^, ^16^ We have previously reported that self-reported laparoscopically-confirmed endometriosis is highly reliable, with 95–100% being confirmed via medical records.^17^ Blood samples were collected from 29,611 participants who were aged 32–54 years old between 1996–1999.^18^ Premenopausal women who had not taken exogenous hormones and who had not breastfed or been pregnant within 6 months provided a blood sample at the mid-luteal phase, 7–9 days before the anticipated start of their next cycle (n = 18,521). Women who declined or were unable to provide a timed sample provided an untimed blood sample (n = 11,090). Blood samples were shipped with an ice-pack via overnight courier and processed using a standard protocol separating into plasma and blood cell components and stored in liquid nitrogen freezers. Participants who provided blood samples completed a questionnaire at time of blood draw that included data on the date and time of day of blood sample collection, current weight, smoking status, medication use, hours since last food intake, and first day of the menstrual cycle in which the blood samples were drawn. Participants subsequently reported the first day of the next menstrual cycle following blood draw.

### Ethics

The study protocol was approved by the Institutional Review Boards (IRBs) of the Brigham and Women’s Hospital and Harvard T.H. Chan School of Public Health. At enrolment, participants received a letter being informed about the intent of the research and the IRBs allowed participants’ completion of the self-administered questionnaire to be considered as implied consent (2019P002978). All participants were allowed to opt out of the study at any time.

### Nested case–control study

For the current study, we selected 200 endometriosis cases diagnosed after blood draw (incident cases) who were premenopausal at time of blood draw and 200 risk-set sampling matched premenopausal controls in the NHSII. Eligibility of endometriosis cases in this study were those who self-reported they had laparoscopically-confirmed endometriosis in their biennial questionnaires, in which we have previously reported 97% validity.^17^ Controls were selected from participants without endometriosis in the same questionnaire cycle, matching on the matching factors. Among the selected incident endometriosis cases, bloods were collected up to nine years prior to endometriosis diagnosis to discover proteins associated with endometriosis. For each endometriosis case, one control was randomly selected from the risk set matched on age (±1 years), race/ethnicity (non-Hispanic white, other race/ethnicity), history of infertility or failure to conceive after one year of trying (ever/never reported), blood sample characteristics (timing of menstrual phase, month (±1 month), time of day (±2 h), and fasting status (<2, 2–4, 5–7, 8–11, ≥12 h) at blood draw). Matching was based on addressing factors most likely to influence biomarker levels but not mediators between the biomarker and outcome. In addition, we selected a testing cohort of 103 incident endometriosis cases and 103 risk-set sampling matched controls with bloods collected up to 4 years prior to endometriosis diagnosis within the NHSII to validate some of the identified proteins using enzyme-linked immunosorbent assay (ELISA). From our experience with proteomic effect on clinical outcomes, the odds ratio of a good proteomic biomarker ranges from 1.5 to 2.0 or higher per 1 standard deviation change. When Type-I error was set at 0.05, we could detect an odds ratio of around 1.4 or higher with a sample size of 155 pairs and odds ratio of 1.5 or higher with a sample size of 107 pairs.^19^ When type-I error was set at 0.000038 adjusting for multiple testing of 1300 proteins, we could detect an odds ratio of 1.7 or higher with our sample size of 200 pairs.

### SomaScan proteomics assay

SomaScan Assay Kit for human plasma 1.3 k v3.1 was used to measure relative protein levels in 55 μL plasma, which we have previously reported has high reproducibility and within-person stability over time.^20^ Using highly selective single-stranded Slow Off-rate Modified DNA Aptamers (SOMAmer), we simultaneously quantified 1305 proteins following the manufacturer’s standard protocol (SomaLogic; Boulder, CO). The SOMAmer reagents have undergone validation experiments for performance and specificity.^21^ Quality control (QC), calibration, and normalisation of the data using pooled plasma samples provided by the manufacturer was conducted following the manufacturer’s protocol as previously described.^22^^,^^23^ Thirty additional QC samples blinded to the laboratory were randomly distributed among the participants’ samples, which showed that 98% of proteins had intra-batch coefficients of variation (CVs) < 25%, 99% had inter-batch CV < 25%; and there were no missing protein values. Of the 1305 proteins measured, proteins with poor stability over time were excluded (n = 568), based on our prior evaluation of delayed processing after blood collection.^20^ This resulted in 773 proteins being included in the final analysis.

### ELISA validation

We conducted ELISA on three nominally significant proteins [insulin like growth factor binding protein 1 (IGFBP1), CD5 antigen-like (CD5L), Annexin A1 (ANXA1)], which had an absolute fold change of at least 1.2 or higher from the analysis of the SomaScan data, had a commercially available ELISA kits, and also required minimal plasma volume (≤100 μL), to validate the performance of the SomaScan assay. Of note, in our previous pilot study,^20^ ANXA1 had intraclass correlation (ICC) of 0.80 when examining bloods immediately processed vs. bloods processed 24 h after collection, but the ICC across blood samples processed immediately, 24 h after collection, and 48 h after collection was 0.57. The IGFBP-1 ELISA assay was run on the fully automated immunoassay Ella instrument (ProteinSimple/Bio-Techne, Inc., Minneapolis, MN) as a single-plex using 72-well cartridges (part# SPCKB-PS- 000327) following the manufacturer’s instructions. The Annexin A1 (catalog# ELH-ANXA1) and CD5L (catalog# ELH-CD5L) ELISA assays were purchased from RayBiotech, Inc. (Peachtree Corners, GA). Each assay was run according to the manufacturers’ recommended specific protocol. An assay specific standard curve was generated on each ELISA plate. Samples with CVs > 15% were repeated and the repeated measurement was used.

### Statistics

Conditional logistic regression models accounting for the matching factors [i.e., age (±1 years), race/ethnicity (non-Hispanic white, other race/ethnicity), history of infertility or failure to conceive after one year of trying (ever/never reported), blood sample characteristics (i.e., timing of menstrual phase, month (±1 month), time of day (±2 h), and fasting status (<2, 2–4, 5–7, 8–11, ≥12 h) at blood draw)] were used to estimate the odds ratios (ORs) and 95% confidence intervals (CIs) per one standard deviation (SD) increase in protein levels. Since this analysis was conducted using a nested case–control study (i.e., nested within a prospective cohort study), the calculated ORs are an estimate of the risk ratio.^24^ The odds ratios can estimate the relative risk well in the case of the outcome having small probability of occurrence. In this study, we report the odds ratios as an estimate of the relative risks as the prevalence of our outcome of interest, endometriosis, is known in the population to be low (about 10–11%).^1^ False discovery rate (FDR) was calculated to account for multiple testing. Fold change of protein levels was calculated by dividing the mean protein level from the case to control within each pair, then taking the mean of these pair-specific ratios. If this ratio was less than one, we inverted the ratio and multiplied by −1.

We conducted analysis restricted to those with blood collected ≤2 years prior to endometriosis diagnosis to assess plasma proteins in blood collected proximal to diagnosis. We used Spearman correlation to examine the correlations between IGFBP1, CD5L, and ANXA1 values measured using SomaScan and ELISA on the same samples. To examine the performance of the multi-protein model in discriminating endometriosis cases from controls, we calculated the area under the curve (AUC). To develop a multiprotein model based on the SomaScan data we selected the nominally significant proteins that had an absolute fold change >1.2.^25^ We restricted the proteins to those that had an absolute fold change >1.2 because if we were to develop a clinically applicable biomarker panel, ideally, we would want to select proteins that have large magnitude of differences between endometriosis cases and controls. We identified the top 3 models with the lowest AICs and highest AUCs for each dimension (based on the score test) for all the protein combinations (i.e., top 3 models with 1 protein, top 3 models with 2 proteins, and so on, until the top 3 models with all 17 proteins). We used the ‘all subset model selection procedure’ to select the best model among these, which is a model selection method based on the ranking of the AIC and AUC from all possible main effect models that could be formed from the combinations of the predictors (proteins).^25^, ^26^, ^27^, ^28^ The smaller the AIC the better fit the model and the larger the AUC, the better the model in terms of discrimination. Thus, the optimal model would have small AIC and large AUC, and the models are ranked based on the AUC and AIC value. Therefore, we looked at the models with the highest AUC and chose the model with the lowest AIC among them.

For protein–protein functional and physical interactions, STRING database version 12.0 was used and the results were displayed as a functional network.^29^ Interactions were considered with a STRING confidence score of ≥0.4 (out of 1.0) garnered from the “experimental” and “databases” categories. Proteins without associations to other proteins in the network were removed. A k-means clustering algorithm was performed to select connected proteins based on the distance matrix obtained from the String global scores. Seven clusters were selected as it provided the most optimal number of proteins per cluster. Databases including Gene Ontology (GO), KEGG, REACTOME, STRING local network cluster terms, and a PubMed literature search was used for determining functional enrichment of the proteins in the clusters. Systems biology analyses were performed using the Ingenuity Pathways Knowledge Base (Qiagen, Redwood City, CA). Ingenuity Pathway Analysis (IPA) was used to perform functional category analysis of all dysregulated proteins with a p-value < 0.05 (Wald test) from the logistic regression analysis, which provides new insights into potential pathophysiological pathways underlying endometriosis-specific plasma protein signatures (QIAGEN, Redwood City, CA).^30^ All statistical analyses were conducted using SAS/STAT 14.2 (SAS Institute Inc. 2014, Cary, NC, USA).

### Role of funders

The funders had no role in study design, data collection, data analyses, interpretation, or writing of report.

## Results

### Study population description

**Fig. 1:**
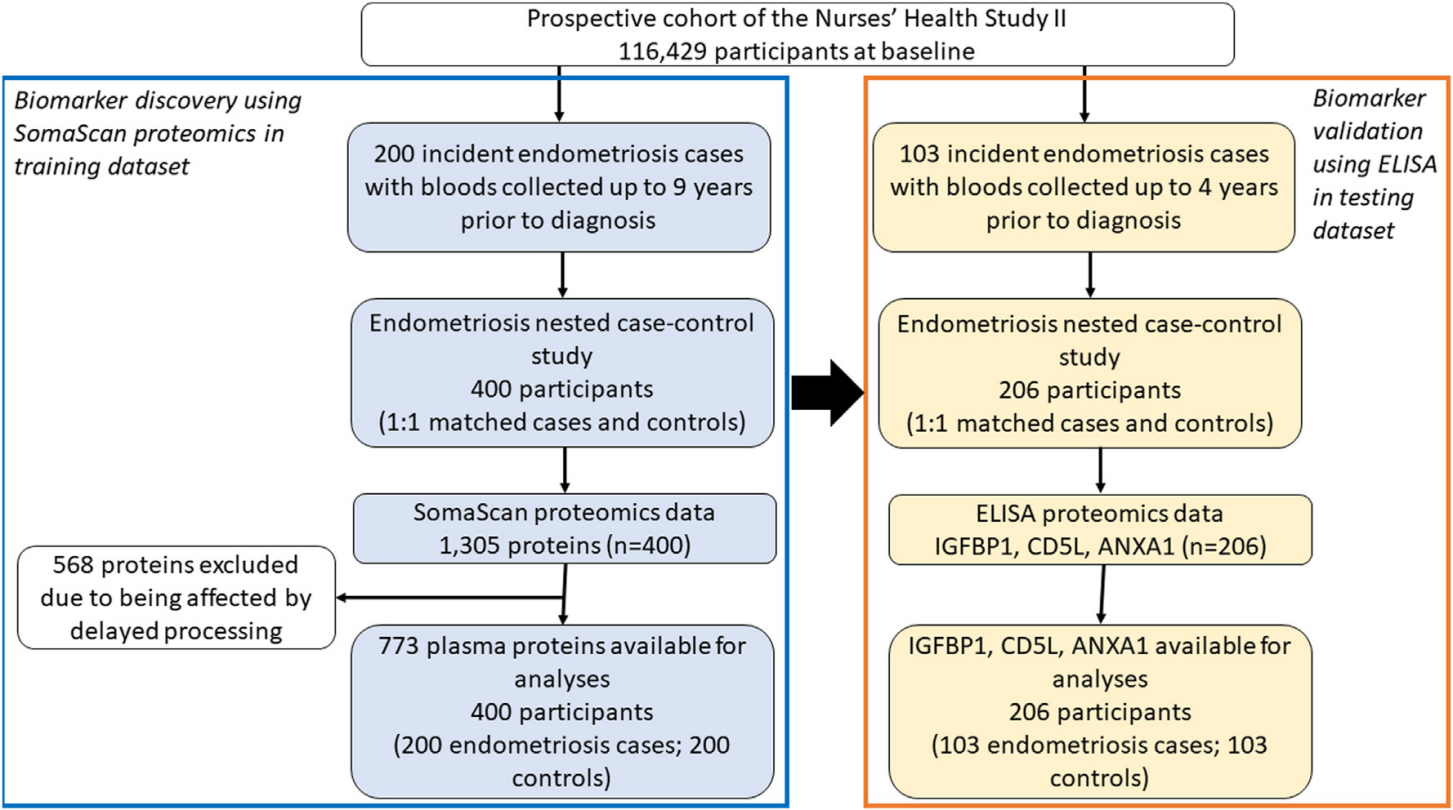
**Overview of study design.** We first measured 1305 plasma proteins using SomaScan on the nested endometriosis case–control study within the NHSII cohort, including 200 endometriosis cases and 200 matched controls. Then, we evaluated three protein markers (i.e., IGFBP1, CD5L, ANXA1) that were identified to be nominally significantly associated with endometriosis using ELISA in an independent set of 103 endometriosis cases and 103 controls nested within the NHSII cohort.

A diagram summarising the overall study design and analytic datasets are shown in Fig. 1. We first measured plasma proteomics using SomaScan on 200 endometriosis cases and 200 matched controls and then evaluated three protein markers that were identified to be associated with endometriosis using ELISA in an independent set of 103 endometriosis cases and 103 controls within the NHSII cohort. The median age at blood draw was 40.7 years for endometriosis cases diagnosed after blood draw (incident) and 41.3 years for risk-set sampling matched controls, and most participants were white race (>95%) with average BMI of 25–26 kg/m^2^ (Table 1). Compared to controls, endometriosis incident cases were more likely to be nulliparous (23% vs. 33%). Blood samples from endometriosis cases were collected up to 9 years before endometriosis diagnosis (median = 4 years before diagnosis).

**Table 1:** Characteristics of incident laparoscopically-confirmed endometriosis cases and matched^a^ controls in the Nurses’ Health Study II.

	Endometriosis cases (n = 200)	Controls (n = 200)
Age at blood draw, years, mean (SD)	40.7 (4.0)	41.3 (3.9)
White Race, n (%)	196 (98)	198 (99)
Body mass index, kg/m2, mean (SD)	26.1 (6.3)	25.7 (6.5)
Cigarette smoking history, n (%)		
Never	146 (73)	140 (70)
Past	37 (19)	38 (19)
Current	17 (9)	22 (11)
Age at menarche, n (%)		
<12 years old	48 (24)	45 (23)
12 years old	64 (32)	60 (30)
>12 years old	88 (44)	95 (48)
Oral contraceptive use, n (%)		
Never	39 (20)	32 (16)
Ever	161 (80)	168 (84)
History of infertility, n (%)	61 (31)	66 (33)
Parity, n (%)		
Nulliparous	66 (33)	46 (23)
1–2	89 (45)	104 (52)
3 or more	45 (23)	50 (25)
Season of blood draw, n (%)		
Winter	29 (15)	35 (18)
Spring	117 (59)	119 (60)
Summer	39 (20)	33 (17)
Autumn	15 (8)	13 (7)
Time of day of blood draw, n (%)		
0 am–7 am	60 (30)	38 (19)
8 am–1 pm	130 (65)	156 (78)
2 pm–11 pm	10 (5)	6 (3)
Luteal days at blood draw, n (%)		
0–5 days	27 (14)	26 (13)
6–7 days	32 (16)	34 (17)
8–9 days	90 (45)	90 (45)
10 days or more	51 (26)	50 (25)
Fasting ≥ 8 h at blood draw, n (%)	125 (63)	159 (80)

a Controls were risk-set sampling matched to incident endometriosis cases on age (±1 years), race/ethnicity (non-Hispanic white, other race/ethnicity), history of infertility (ever/never reported), blood sample characteristics (i.e., timing of menstrual phase, month (±1 month), time of day (±2 h), and fasting status (<2, 2–4, 5–7, 8–11, ≥12 h) at blood draw). Luteal days are presented for those who had timed blood samples. Percentages may not sum to 100% due to rounding.

### Individual proteins associated with endometriosis risk

**Fig. 2:**
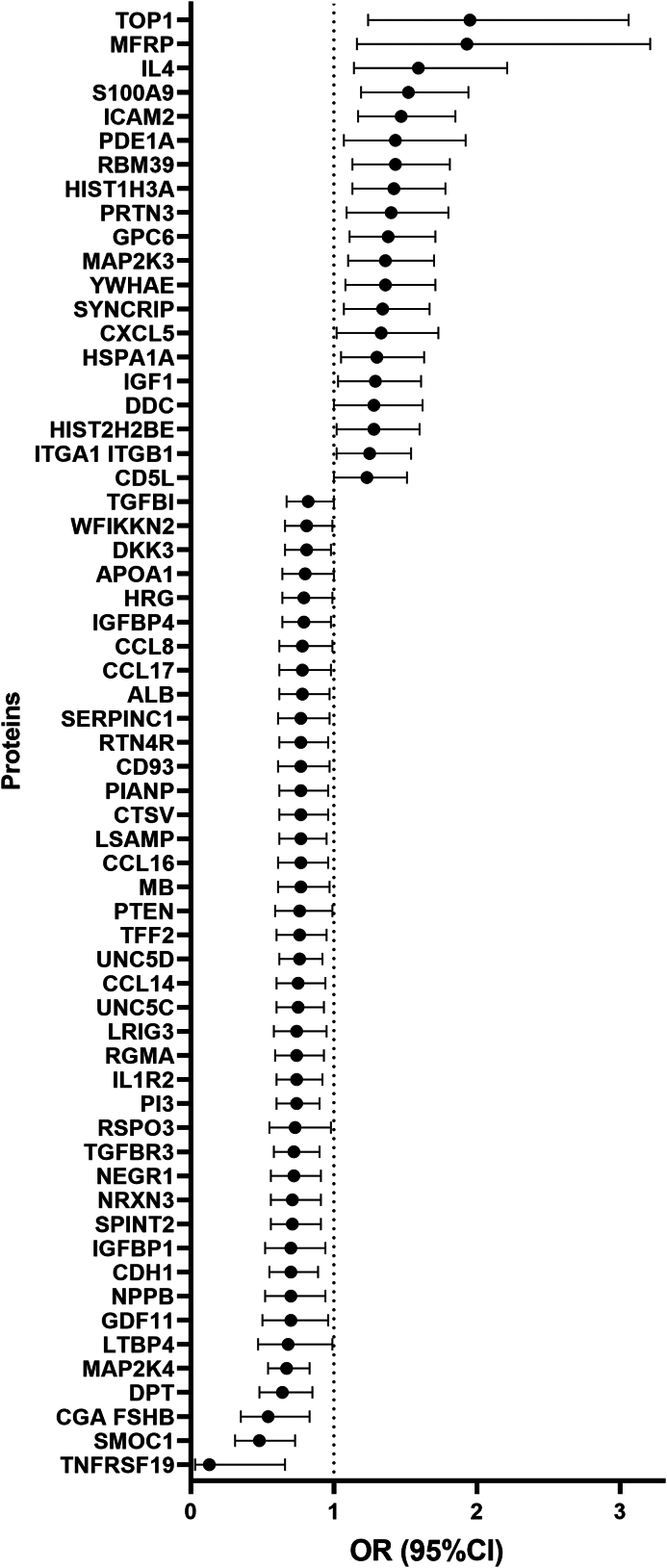
**Plasma proteins associated with endometriosis comparing 200 cases diagnosed at least two years after blood draw and 200 risk-set sampling matched controls in NHSII with nominal p < 0.05[Wald test].** There were 61 proteins that were associated with endometriosis, in which the individual protein names are listed in the y axis using the Entrez Gene Symbol, and Odds Ratio (95% Confidence Intervals) plotted on the x axis.

**Fig. 3:**
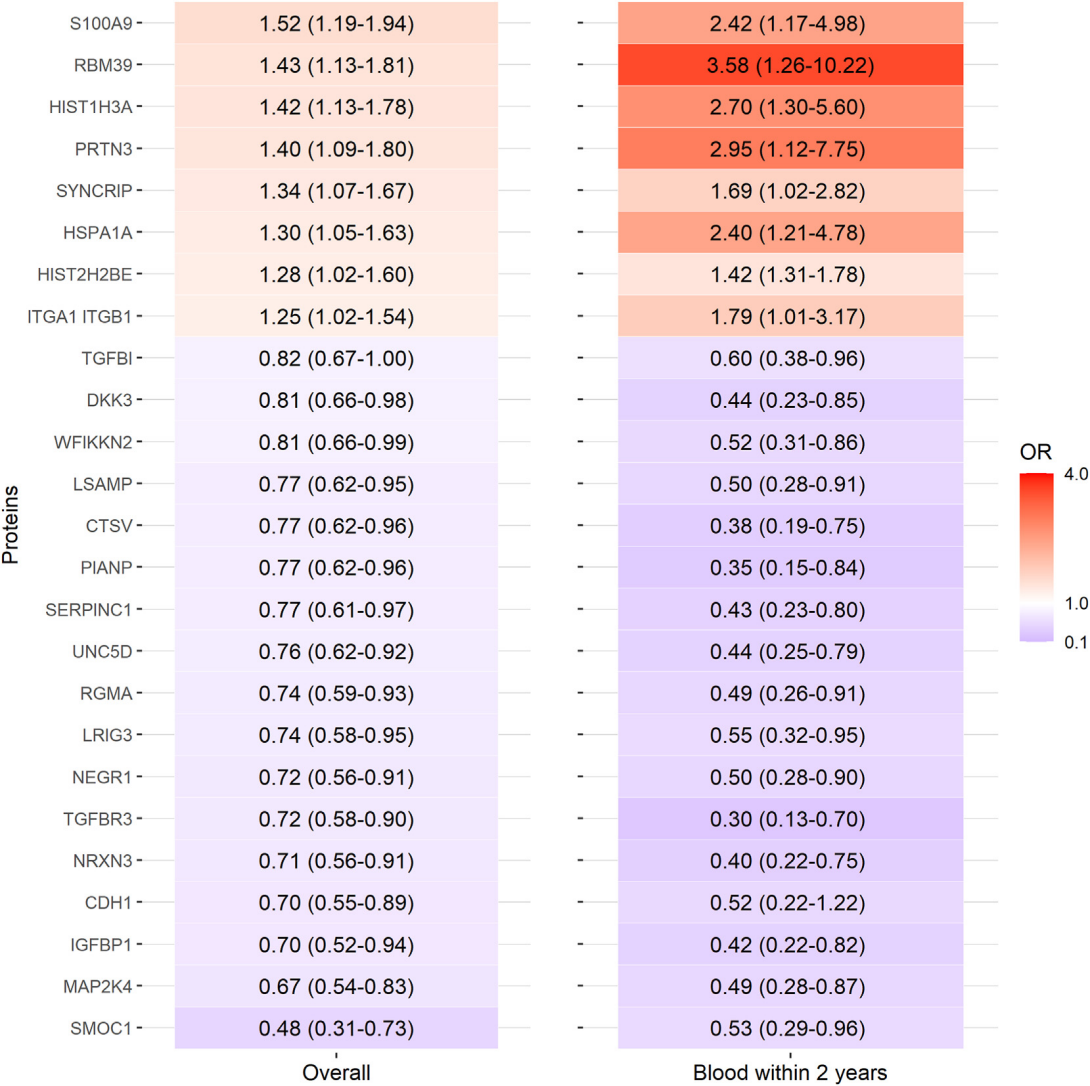
**Proteins associated with endometriosis in both overall (left column) and in blood collected ≤2 years prior to endometriosis diagnosis (right column) among the 61 proteins with nominal p < 0.05[Wald test] in the overall analysis.** There were 25 proteins that were nominally significantly associated with endometriosis in both the overall analysis and when restricted to those with bloods collected proximal to endometriosis diagnosis. Individual protein names are listed in the y axis using the Entrez Gene Symbol. The numbers in the boxes indicate the Odds Ratios (95% Confidence Intervals).

When examining proteins individually, 61 proteins were nominally significantly associated with risk of endometriosis diagnosis overall (nominal p < 0.05 [Wald test]; Fig. 2, Supplementary Table S1). Considering in the order of significance, compared to controls, endometriosis incident cases had higher plasma levels of DNA topoisomerase I (TOP1; OR = 1.95, 95%CI = 1.24–3.06), Protein S100-A9 (S100A9; OR = 1.52, 95%CI = 1.19–1.94), Intercellular adhesion molecule 2 (ICAM2; OR = 1.47, 95%CI = 1.17–1.85), Histone H3.1 (HIST1H3A; OR = 1.42, 95%CI = 1.13–1.78), CD5L (OR = 1.23, 95%CI = 1.004–1.51), that resulted in greater odds of endometriosis, and lower levels of IGFBP1 (OR = 0.70, 95%CI = 0.52–0.94), Neuronal growth regulator 1 (NEGR1; OR = 0.72, 95%CI = 0.56–0.91), and Neurexin-3-beta (NRXN3; OR = 0.71, 95%CI = 0.56–0.91) associated with reduced odds of endometriosis. When we restricted to those who had blood drawn proximal to their endometriosis diagnosis (≤2 years prior), we generally observed greater magnitude of associations (Fig. 3, Supplementary Table S2). For example, the association between S100A9 levels and endometriosis risk was OR = 1.52 (95%CI = 1.19–1.94) in the overall analysis but the magnitude of association increased to OR = 2.42 (95%CI = 1.17–4.98) when examining those who had blood collected proximal to endometriosis diagnosis.

### Analytic validation of nominally significant proteins in an independent sample

**Fig. 4:**
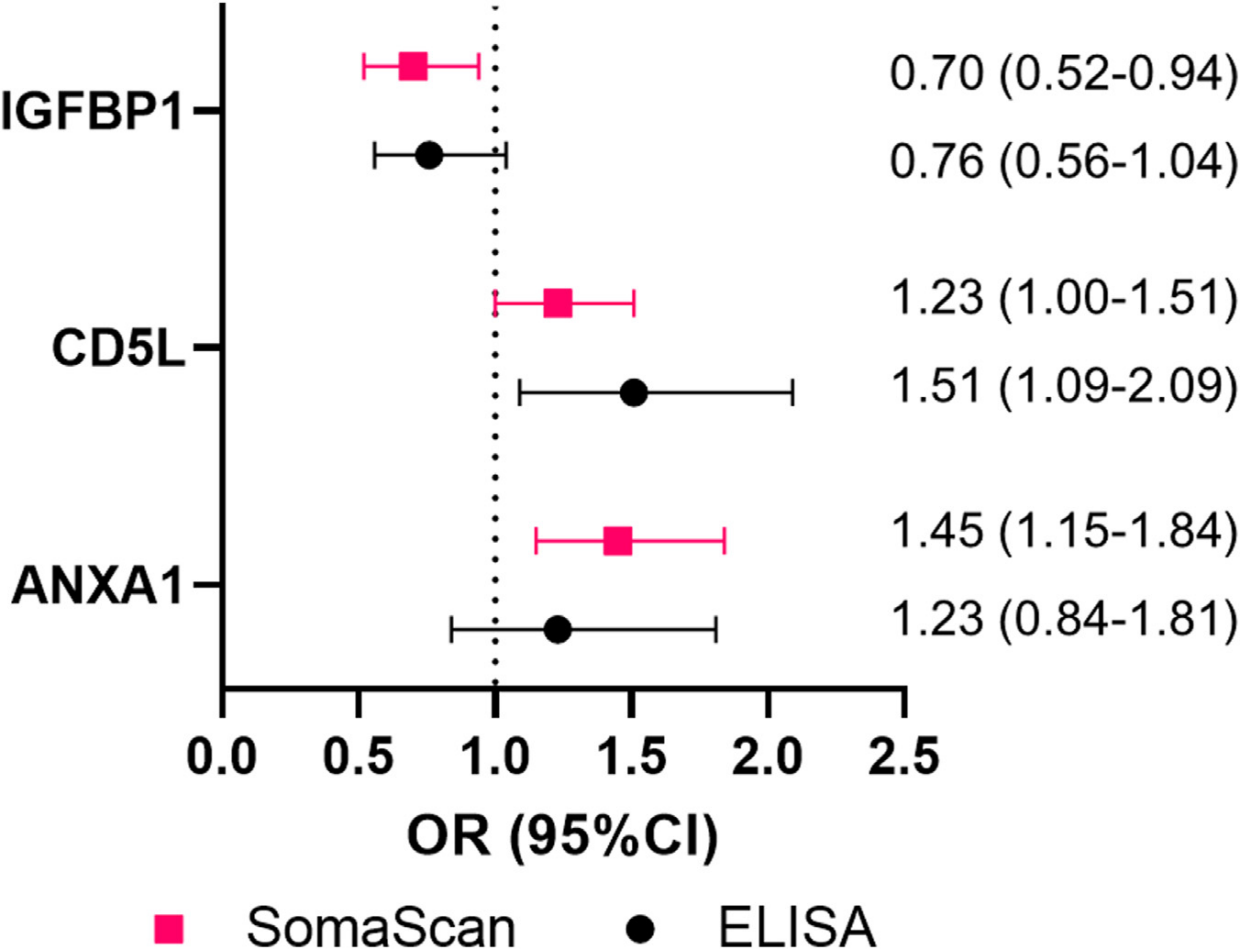
**Independent validation of protein markers identified using SomaScan assay results.** We measured IGFBP1, CD5L and ANXA1 using ELISA and examined its association with endometriosis risk in an independent dataset of 103 endometriosis cases and 103 risk-set sampling matched controls nested within the Nurses’ Health Study II to validate our findings from the SomaScan assay results, which were based on 200 endometriosis cases diagnosed after blood collection and 200 risk-set sampling matched controls nested within the Nurses’ Health Study II.

We used ELISA as an orthogonal technology to measure the absolute expression levels of three protein markers (i.e., IGFBP1, CD5L, ANXA1) in which the SomaScan analysis results showed nominally significant association with absolute fold change of >1.2 between endometriosis cases and controls and ELISA assays were commercially available. To examine the correlations between SomaScan measurements and ELISA measurements, we measured IGFBP1 on 44 samples that also had SomaScan data, 32 samples for CD5L, and 27 samples for ANXA1. The Spearman correlation coefficients were 0.88 for IGFBP1, 0.50 for CD5L, and −0.46 for ANXA1. When we tested the associations between these three protein markers measured using ELISA and endometriosis risk in an independent dataset of 103 incident endometriosis cases and 103 risk-set sampling matched controls in the NHSII, we observed the direction of associations were the same as those observed in the SomaScan analysis for all three markers (Fig. 4).

### Multi-protein model discriminating endometriosis cases from controls

We developed a multiprotein model from the SomaScan data to assess its performance in discriminating endometriosis cases from controls in bloods collected years prior to endometriosis diagnosis. The multiprotein models were developed based on 17 of the 61 nominally significant proteins that had an absolute fold change >1.2 (Supplementary Table S3). The model with the lowest AIC and highest AUC was a 4-protein model including TOP1, HIST1H3A, IGFBP1, and CD5L which discriminated endometriosis cases from controls with an AUC = 0.71 (95%CI = 0.66–0.76) from the conditional logistic regression model. When we additionally adjusted for nulliparity in the 4-protein model, the effect estimates remained similar, suggesting nulliparity is not a strong confounder.

### Biological pathway analysis

**Fig. 5:**
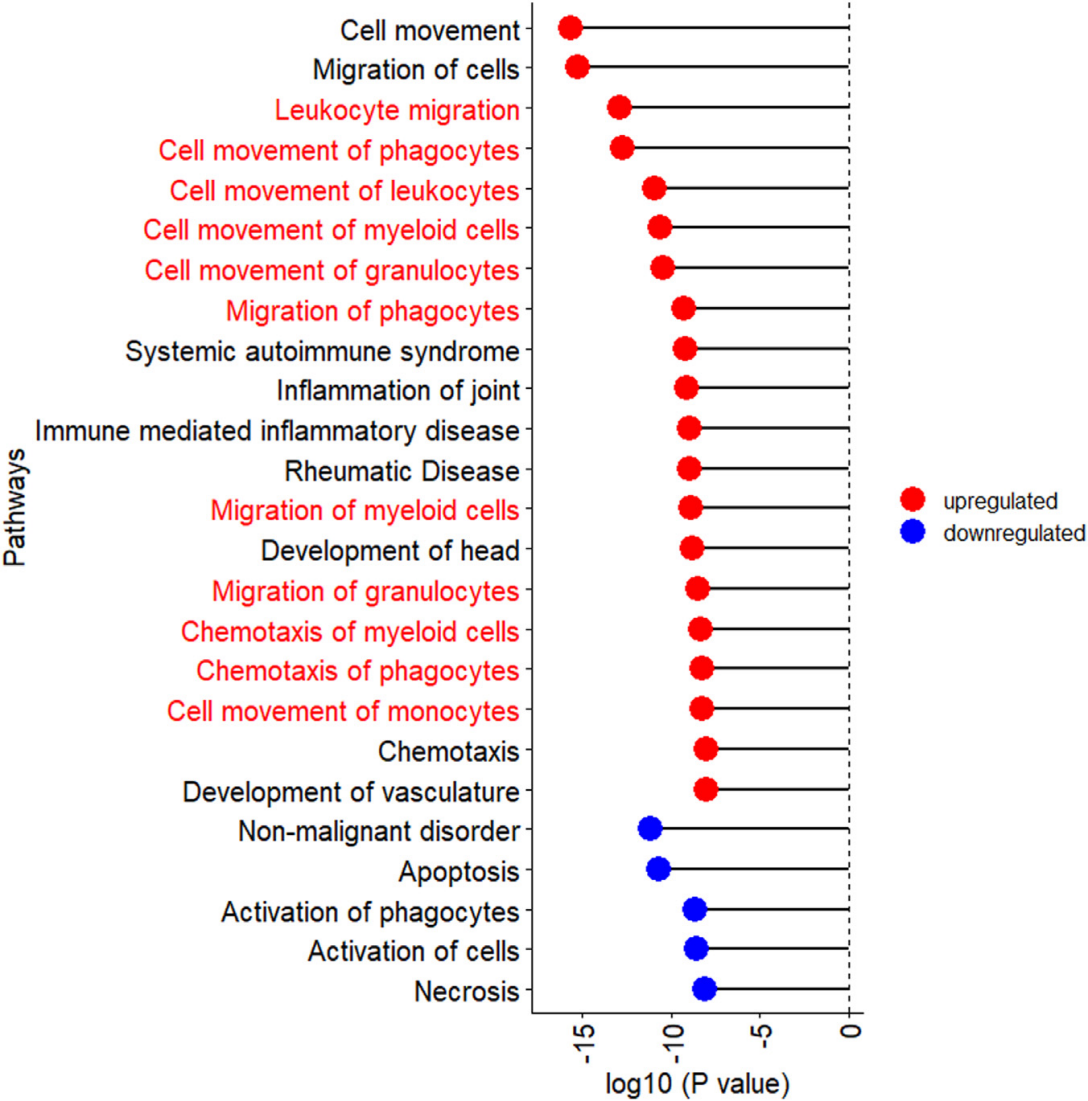
**Biological pathways associated with risk of endometriosis diagnosis (n = 400, NHSII).** The top 25 significant biological pathways associated with risk of endometriosis diagnosis in which the activation z-scores is not zero, are grouped by the direction of association (i.e., activation z-score) and presented within group ordered by p-value (Wald test). Upregulated pathways are denoted by red bubbles and downregulated pathways are denoted by blue bubbles. Pathways related to upregulation of innate immune cell migration are in red font.

**Fig. 6:**
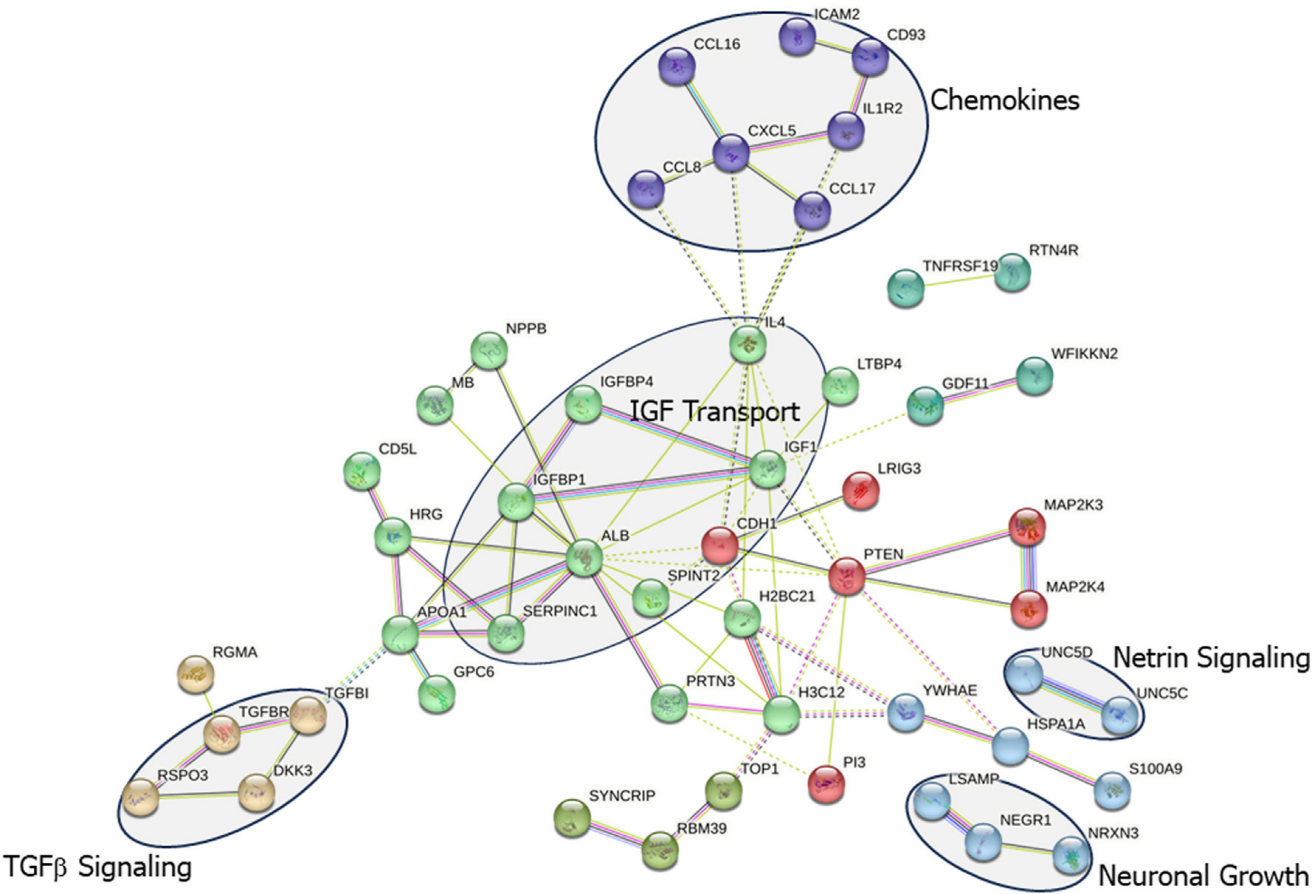
**Protein–protein interaction clusters and relevant pathways associated with risk of endometriosis diagnosis using STRING analysis (k-means = 7 clusters indicated by node colour; n = 400, NHSII).** Protein–protein interaction network was created based on the 61 proteins that were nominally significantly associated with risk of endometriosis diagnosis in the Nurses’ Health Study II. Related functional categories are labelled based on proteins with their reported functional involvement in the pathways of insulin-like growth factor (IGF) transport (green node), tumour growth factor (TGF)β signalling (yellow node), chemokines (purple node), and neuronal growth and netrin signalling (light blue node). Solid line represents within-cluster, dashed grey line represents between-cluster interactions. Line thickness indicates strength of data support.

Multiple immune-related pathways identified by Ingenuity Pathway Analysis were upregulated in bloods collected up to 9 years prior to endometriosis diagnosis (Fig. 5, Supplementary Table S4). Interestingly, multiple pathways related to upregulation of cell movement/migration of cells in the myeloid lineage such as granulocytes, phagocytes, and myeloid cells were upregulated, suggesting activation of innate immunity. Upregulation of other pathways related to immune-related diseases were observed, such as systemic autoimmune syndrome, immune mediated inflammatory disease, and rheumatic diseases. These results support that women and girls with endometriosis present with systemic immune dysregulation years prior to being clinically diagnosed with endometriosis. These pathway findings were also supported by the individual protein–protein interactions among the 61 nominally significant proteins. We observed clusters with groups of proteins related to IGF transport, TGFβ signalling, chemokines, neuronal growth, and netrin signalling (Fig. 6). Interestingly, proteins related to neuronal growth and netrin signalling were also observed.

## Discussion

While dysregulation of systemic inflammation/immunity has been observed in many cross-sectional studies examining blood samples collected from prevalent endometriosis cases (diagnosis was before blood draw), our results indicating upregulation of systemic innate immune response years prior to endometriosis diagnosis suggests endometriosis is a systemic disease with similarities to autoimmune disease and that immune system dysregulation plays a role in endometriosis development.

The individual protein and biological pathway results show activation of systemic innate immunity present years prior to clinical diagnosis of endometriosis. In particular, IGF1 is a protein hormone that plays a prominent role in the regulation of immunity and inflammation.^31^ IGF1 enhances innate immunity by promoting proliferation and differentiation of myeloid cells and promotes anti-apoptotic signalling, enhancing survival of these cells. IGFBPs bind to IGFs and regulate the bioavailability of IGFs in circulation.^32^ Our results showing IGF1 increasing and IGFBP1 and IGFBP4 reducing the odds for an endometriosis diagnosis suggest an opposing function of IGF1 and their binding proteins in bloods collected years prior to endometriosis diagnosis. Our results are in line with a prior study in the NHSII population showing that blood IGF1 levels were positively associated with endometriosis risk among younger women (age<40 years at blood draw),^33^ and adds further insight into the additional proteins that contribute to the IGF transport pathway. CD5L is a 40 kDa soluble glycoprotein and innate immune effector that is produced mainly by macrophages^34^ in lymphoid and inflamed tissue. CD5L influences monocyte inflammatory response and regulates innate immune signalling. TOP1 is involved in the transcriptional response to pathogens and plays a pivotal role in innate immune response.^35^ When TOP1 activity is inhibited, the inflammatory response to various pathogens is compromised. Additionally, certain chemical compounds that inhibit TOP1 have been observed to suppress the expression of genes that promote inflammation. CXCL5 has been reported to play a critical role in regulating innate immune response.^36^ IL4 plays a key role in Th-2 mediated immune response.^37^^,^^38^ Produced by activated CD4^+^ T cells, IL4 affects innate immune cells, polarises macrophages to M2-like phenotype and enhances allergic inflammation.^39^^,^^40^ S100A9 is a protein that is expressed and released by neutrophils, monocytes, and activated macrophages together with S100A8.^41^ The S100A8/9 heterodimers have been shown to signal via Toll-like receptor 4 and regulate innate inflammatory response. Higher levels of S100A8/9 levels have been correlated with disease progression in patients’ with early osteoarthritis, and has been reported to be an important mediator of pain response during the acute phase of inflammation in arthritis mouse models.^42^ As we observed that the magnitude of association increased between S100A9 and endometriosis in bloods collected closer to endometriosis diagnosis, it is plausible that S100A9 contributes to early endometriosis aetiology and pain symptomatology.

Our results showing systemic immune dysregulation years prior to endometriosis diagnosis is in line with epidemiological literature showing consistent associations between endometriosis and autoimmune diseases. Endometriosis and autoimmune diseases have been observed to coexist in many cross-sectional studies.^43^ However, whether endometriosis is a risk factor for autoimmune diseases or if endometriosis is a consequence of autoimmune diseases, and whether endometriosis and autoimmune disease share similar pathophysiological mechanisms remains unclear.^44^ Our results suggest that systemic immune dysregulation is a risk factor for endometriosis that presents years prior to clinical diagnosis of endometriosis. In fact, some of the proteins we identified have also been associated with autoimmune conditions, such as S100A9 and rheumatoid arthritis,^45^ CD5L and systemic lupus erythematosus.^46^ Furthermore, these autoimmune diseases are more common women, accounting for 80% of autoimmune diagnoses.^47^ Hormonal differences and a more robust immune response have been posited to play a role in the higher risk in women^48^ and may also contribute to endometriosis aetiology. Further research is needed to understand the potential shared aetiology of endometriosis and autoimmune disease.

Several proteins related to TGFβ signalling pathway were negatively associated with endometriosis in our analysis, although prior cross-sectional studies reported TGFβ1 ligands and signalling molecules to be higher in blood of those diagnosed with endometriosis compared to those without.^49^ This suggests that upregulation of TGFβ signalling pathway may be a consequence of endometriosis disease rather than contributing to disease development.

Interestingly, several proteins related to neurological disorders were identified to be associated with endometriosis in our analysis. For example, NRXN3 has been associated with fibromyalgia^50^ and headaches,^51^ and is known to act in the nervous system as a receptor and cell adhesion molecule. NEGR1 plays a role in axon extension, synaptic plasticity, synapse formation, and processes key neuronal functioning.^52^, ^53^, ^54^ Consistent with our finding, one study previously reported lower expression of UNC5C and UNC5D in endometriotic lesions compared to eutopic tissue of those without endometriosis.^55^ Netrin-1 is an axon guide molecule and has been observed to be elevated in patients with endometriosis and positively correlated with endometriosis-associated pain^55^. Netrin-1 is thought to mediate endometriosis-associated pain by promoting nerve fibre infiltration in endometriotic lesions.

We have previously conducted a cross-sectional analysis and reported plasma proteomics profiles associated with endometriosis diagnosed primarily in adolescents and young adults compared to controls.^23^ Interestingly, there were minimal overlap between the results observed in the current study and individual proteins associated with endometriosis diagnosed in a young patient population. While this may in part be due to the current study only being able to evaluate 773 proteins out of 1305 proteins measured as the blood samples in NHSII were all affected by delayed processing, this difference may also be due differences in the types of endometriosis represented in these two studies. Endometriosis cases in NHSII were diagnosed in adulthood, and therefore are more likely to be presenting with infertility and deep and/or endometriomas whereas our prior study included adolescents and young adults diagnosed with endometriosis who presented with severe pelvic pain and superficial peritoneal lesions only.

This study has several strengths, as well as some limitations. This study agnostically examines a large panel of circulating proteins in prospectively collected blood samples of women with and without endometriosis. No other human study has captured this pre-diagnostic window in endometriosis, which is temporally valid for causal inference potentially capturing the actual etiologic window.^1^ While immune dysregulation has been reported to be associated with endometriosis, most of the studies used blood samples collected from patients already diagnosed with endometriosis, in which the blood proteomic profiles are likely impacted by having the disease itself. Thus, our study provides population-level data indicating that systemic immune dysregulation is occurring prior to endometriosis diagnosis, providing additional evidence supporting its role in endometriosis disease development. While validation of the model in an independent prospective cohort setting is necessary, and generalisability may be limited to endometriosis cases similar to those included in this study (older incident diagnosis cases due to age at blood draw within this cohort and primarily white), we identified candidate proteins that may distinguish endometriosis cases from controls for earlier diagnosis. Our results showing greater magnitude of associations in bloods drawn proximal to endometriosis diagnosis further supports the feasibility of a blood-based biomarker for earlier diagnosis of endometriosis. Nonetheless, further research is needed to decipher whether these changes in magnitude were due to the presence of sub-clinical endometriosis rather than reflective of an endometriosis etiologic protein profile.

We also used a reproducible proteomics technology to measure plasma proteins. While SomaScan assay results have generally had high correlations with absolute concentrations measured using ELISA,^56^ we were able to analytically validate a few proteins identified by SomaScan using ELISA and replicated the association with endometriosis in an internal independent validation dataset. However, no external validation dataset was available for further replication. Interestingly, while ANXA1 values measured in SomaScan did not correlate with ANXA1 values measured in ELISA, we did observe similar direction of association with endometriosis risk. The low correlation implies that SomaScan assay and ELISA are measuring different proteoforms of the ANXA1 protein, but regardless they both seem to capture ANXA1 that is positively associated with endometriosis risk. Further protein validation research is needed to elucidate this observation.

The observed AUC of the developed multi-protein model was modest, therefore further research is needed to expand the model to achieve higher AUC and to validate in an external dataset in order for the model to be clinically applicable. Since the blood samples were collected before the endometriosis disease status was known and the lab was blinded to endometriosis case/control status, we would anticipate any measurement error of plasma protein levels to be non-differential misclassification and would bias towards the null. Since the menstrual cycle phase was determined based on self-reported cycle regularity and the day of one’s first and next menstrual cycle phase, misclassification is possible. The NHSII did not have information on revised American Society of Reproductive Medicine (rASRM) stage and therefore we were not able to examine similarity or differences across rASRM stage. We were not able to evaluate the associations by surgical macrophenotype as we have confirmed that these data are not available systematically in medical record documentation.^57^^,^^58^ As we have matched on various factors, although most factors being related to blood collection, it is possible that we may have underestimated the effects of proteins that are highly correlated with the matching factors. We acknowledge that we had modest sample size and power to identify individual proteins associated with endometriosis. We acknowledge that there was insufficient sample size to perform subgroup analyses, such as by parity and BMI. However, to our knowledge, this study is the largest prospective study to date that has comprehensively evaluated proteomics in blood samples collected years prior to endometriosis diagnosis. Since this is an observational study, there is a possibility of residual or unmeasured confounding. While the current study investigated plasma proteins measured prior to endometriosis diagnosis providing insight into endometriosis aetiology, we have ongoing parallel studies examining biomarkers in blood samples collected from cohorts of patients diagnosed with endometriosis, which will provide the opportunity to investigate the molecular heterogeneity of endometriosis.

In summary, this unique prospective study examining proteomic profiles in blood samples collected up to 9 years prior to endometriosis diagnosis demonstrated multiple proteins related to innate immune response are upregulated years before endometriosis diagnosis. Our results provide evidence that could lead to risk stratification to enhance earlier diagnosis or potential new therapeutic targets for prevention and earlier intervention with replication of these results in external databases and mechanistic studies in cell lines and animal models. While replication of the results in larger independent datasets is necessary, our findings provide evidence supporting immune dysregulation as a key element of endometriosis pathogenesis.

## Contributors

All authors read and approved the final version of the manuscript. Conceptualisation: NS, SAM, TAL, KLT; Methodology: LHN, AFV, STD, TAL; Data generation: STD, TAL; Visualisation: NS; Funding acquisition: TAL, KLT; Supervision: SAM, TAL, KLT; Writing—original draft: NS; Writing—review & editing: NS, LHN, AFV, STD, MA, ALS, SAM, TAL, KLT. LHN and AFV accessed and verified the data.

## Data sharing statement

Because of participant confidentiality and privacy concerns, data cannot be shared publicly and requests to access NHSII data must be submitted in writing. According to standard controlled access procedures, applications to use NHSII resources will be reviewed by our External Collaborations Committee to verify that the proposed use maintains the protection of the privacy of participants and the confidentiality of the data. Investigators wishing to use NHSII data are asked to submit a brief description of the proposed project [go to https://www.nurseshealthstudy.org/researchers (contact email: nhsaccess@channing.harvard.edu) for details].

## Declaration of interests

Naoko Sasamoto received grant funding from the Department of Defence, National Institutes of Health, American Cancer Society, and the Rivkin Foundation. Long H. Ngo received funding from the Department of Defence. Simon T. Dillon received funding from the Department of Defence. Amy L. Shafrir received funding from Eunice Kennedy Shriver National Institute of Child Health and Human Development (R21 HD107515) and Merrimack College Faculty Development grant. Stacy A. Missmer received grant funding from AbbVie, National Institutes of Health, Department of Defence, and Marriott Family Foundation; received honoraria from WERF, Huilun Shanghai, and University of Kansas Medical Center; travel support for SRI, ESHRE, FWGBD, University of Michigan, MIT, ASRM, Taiwan Endometriosis Society, SEUD, Japan Endometriosis Society, NASEM, Endometriosis Foundation of America, Gedeon Richter Symposium at ESHRE; Board member of AbbVie, Roche, LIDEA Registry, NextGen Jane, Editor of Frontiers in Reproductive Health, Roundtable participation for Abbott, Statistical Advisory Board for Human Reproduction; leadership role in Society for Women’s Health Research, World Endometriosis Society, World Endometriosis Research Foundation, ASRM, ESHRE. Towia A. Libermann received grant funding from the Department of Defence. The remaining authors have no disclosures relevant to this manuscript.
