# Supplementary material for: Prospective evaluation of plasma proteins in relation to surgical endometriosis diagnosis in the Nurses’ Health Study II
Source: eBioMedicine. 2025 Apr 11;115:105688. doi: 10.1016/j.ebiom.2025.105688 (PMC12013659; doi:10.1016/j.ebiom.2025.105688)
Supplement: Supplementary Tables S1–S4 [file mmc1.docx]

| **Supplementary Table S1. Plasma proteins associated with endometriosis risk in the Nurses’ Health Study II (n=400)** | | | | | | |
| --- | --- | --- | --- | --- | --- | --- |
| **Protein** | **Entrez Gene Symbol** | **UniProt ID** | **SomaId** | **Fold change** | **Odds ratio (95%CI)** | **p-value** |
| Dual specificity mitogen-activated protein kinase kinase 4 | MAP2K4 | P45985 | SL007237 | -1.04 | 0.67 (0.54-0.83) | 0.0004 |
| SPARC-related modular calcium-binding protein 1 | SMOC1 | Q9H4F8 | SL011888 | -1.04 | 0.48 (0.31-0.73) | 0.001 |
| Protein S100-A9 | S100A9 | P06702 | SL004477 | 1.40 | 1.52 (1.19-1.94) | 0.001 |
| Intercellular adhesion molecule 2 | ICAM2 | P13598 | SL003177 | 1.09 | 1.47 (1.17-1.85) | 0.001 |
| Dermatopontin | DPT | Q07507 | SL008178 | -1.04 | 0.64 (0.48-0.85) | 0.002 |
| Histone H3.1 | HIST1H3A | P68431 | SL008158 | 1.64 | 1.42 (1.13-1.78) | 0.002 |
| Cadherin-1 | CDH1 | P12830 | SL000055 | -1.03 | 0.70 (0.55-0.89) | 0.003 |
| RNA-binding protein 39 | RBM39 | Q14498 | SL011535 | 2.26 | 1.43 (1.13-1.81) | 0.003 |
| Elafin | PI3 | P19957 | SL004458 | 1.01 | 0.74 (0.60-0.90) | 0.003 |
| Glypican-6 | GPC6 | Q9Y625 | SL012881 | 1.08 | 1.38 (1.11-1.71) | 0.004 |
| DNA topoisomerase 1 | TOP1 | P11387 | SL004305 | 1.35 | 1.95 (1.24-3.06) | 0.004 |
| Transforming growth factor beta receptor type 3 | TGFBR3 | Q03167 | SL005059 | -1.02 | 0.72 (0.58-0.90) | 0.004 |
| Dual specificity mitogen-activated protein kinase kinase 3 | MAP2K3 | P46734 | SL007242 | 1.15 | 1.36 (1.10-1.70) | 0.005 |
| Follicle stimulating hormone | CGA FSHB | P01215, P01225 | SL000428 | 1.09 | 0.54 (0.35-0.83) | 0.005 |
| Kunitz-type protease inhibitor 2 | SPINT2 | O43291 | SL001897 | 1.00 | 0.71 (0.56-0.91) | 0.006 |
| Neurexin-3-beta | NRXN3 | Q9HDB5 | SL008728 | -1.02 | 0.71 (0.56-0.91) | 0.006 |
| Netrin receptor UNC5D | UNC5D | Q6UXZ4 | SL005231 | -1.00 | 0.76 (0.62-0.92) | 0.006 |
| Interleukin-4 | IL4 | P05112 | SL000480 | 1.11 | 1.59 (1.14-2.21) | 0.007 |
| Interleukin-1 receptor type 2 | IL1R2 | P27930 | SL000145 | -1.02 | 0.74 (0.60-0.92) | 0.007 |
| Neuronal growth regulator 1 | NEGR1 | Q7Z3B1 | SL008810 | -1.03 | 0.72 (0.56-0.91) | 0.007 |
| Myeloblastin | PRTN3 | P24158 | SL004008 | 1.49 | 1.40 (1.09-1.80) | 0.008 |
| 14-3-3 protein epsilon | YWHAE | P62258 | SL004984 | 1.12 | 1.36 (1.08-1.71) | 0.008 |
| Heterogeneous nuclear ribonucleoprotein Q | SYNCRIP | O60506 | SL007022 | 1.09 | 1.34 (1.07-1.67) | 0.009 |
| Repulsive guidance molecule A | RGMA | Q96B86 | SL010467 | -1.02 | 0.74 (0.59-0.93) | 0.01 |
| Netrin receptor UNC5C | UNC5C | O95185 | SL005230 | -1.02 | 0.75 (0.60-0.93) | 0.01 |
| Membrane frizzled-related protein | MFRP | Q9BY79 | SL010466 | 1.26 | 1.93 (1.16-3.21) | 0.01 |
| C-C motif chemokine 14 | CCL14 | Q16627 | SL003329 | 1.00 | 0.75 (0.60-0.94) | 0.01 |
| Tumor necrosis factor receptor superfamily member 19 | TNFRSF19 | Q9NS68 | SL004863 | -1.01 | 0.13 (0.03-0.66) | 0.01 |
| Calcium/calmodulin-dependent 3',5'-cyclic nucleotide phosphodiesterase 1A | PDE1A | P54750 | SL011400 | 1.30 | 1.43 (1.07-1.92) | 0.02 |
| Leucine-rich repeats and immunoglobulin-like domains protein 3 | LRIG3 | Q6UXM1 | SL010464 | -1.00 | 0.74 (0.58-0.95) | 0.02 |
| Limbic system-associated membrane protein | LSAMP | Q13449 | SL005196 | -1.02 | 0.77 (0.62-0.95) | 0.02 |
| Insulin-like growth factor-binding protein 1 | IGFBP1 | P08833 | SL000462 | 1.52 | 0.70 (0.52-0.94) | 0.02 |
| Trefoil factor 2 | TFF2 | Q03403 | SL002602 | 1.01 | 0.76 (0.60-0.95) | 0.02 |
| Cathepsin L2 | CTSV | O60911 | SL006910 | 1.02 | 0.77 (0.62-0.96) | 0.02 |
| N-terminal pro-BNP | NPPB | P16860 | SL002785 | 1.34 | 0.70 (0.52-0.94) | 0.02 |
| Heat shock 70 kDa protein 1A | HSPA1A | P0DMV8 | SL000451 | 1.23 | 1.30 (1.05-1.63) | 0.02 |
| C-C motif chemokine 16 | CCL16 | O15467 | SL003300 | 1.06 | 0.77 (0.61-0.96) | 0.02 |
| PILR alpha-associated neural protein | PIANP | Q8IYJ0 | SL019019 | 1.01 | 0.77 (0.62-0.96) | 0.02 |
| Reticulon-4 receptor | RTN4R | Q9BZR6 | SL005208 | -1.00 | 0.77 (0.62-0.96) | 0.02 |
| Myoglobin | MB | P02144 | SL000164 | -1.02 | 0.77 (0.61-0.97) | 0.02 |
| Complement component C1q receptor | CD93 | Q9NPY3 | SL007696 | -1.01 | 0.77 (0.61-0.97) | 0.03 |
| Insulin-like growth factor I | IGF1 | P05019 | SL000047 | 1.08 | 1.29 (1.03-1.61) | 0.03 |
| Serum albumin | ALB | P02768 | SL000254 | -1.01 | 0.78 (0.62-0.97) | 0.03 |
| Antithrombin-III | SERPINC1 | P01008 | SL000272 | -1.01 | 0.77 (0.61-0.97) | 0.03 |
| Growth/differentiation factor 11 | GDF11 | O95390 | SL004345 | -1.02 | 0.70 (0.50-0.96) | 0.03 |
| Insulin-like growth factor-binding protein 4 | IGFBP4 | P22692 | SL005171 | 1.00 | 0.79 (0.64-0.98) | 0.03 |
| C-C motif chemokine 17 | CCL17 | Q92583 | SL003196 | 1.10 | 0.78 (0.62-0.98) | 0.03 |
| Dickkopf-related protein 3 | DKK3 | Q9UBP4 | SL009412 | -1.00 | 0.81 (0.66-0.98) | 0.03 |
| C-X-C motif chemokine 5 | CXCL5 | P42830 | SL003169 | 1.16 | 1.33 (1.02-1.73) | 0.03 |
| Integrin alpha-I: beta-1 complex | ITGA1 ITGB1 | P56199, P05556 | SL003179 | 1.38 | 1.25 (1.02-1.54) | 0.04 |
| Histone H2B type 2-E | HIST2H2BE | Q16778 | SL014983 | 1.78 | 1.28 (1.02-1.60) | 0.04 |
| Histidine-rich glycoprotein | HRG | P04196 | SL006448 | 1.03 | 0.79 (0.64-0.99) | 0.04 |
| WAP, Kazal, immunoglobulin, Kunitz and NTR domain-containing protein 2 | WFIKKN2 | Q8TEU8 | SL010391 | 1.02 | 0.81 (0.66-0.99) | 0.04 |
| R-spondin-3 | RSPO3 | Q9BXY4 | SL018509 | -1.00 | 0.73 (0.55-0.98) | 0.04 |
| Latent-transforming growth factor beta-binding protein 4 | LTBP4 | Q8N2S1 | SL007033 | -1.02 | 0.68 (0.47-0.99) | 0.04 |
| Phosphatidylinositol 3,4,5-trisphosphate 3-phosphatase and dual-specificity protein phosphatase PTEN | PTEN | P60484 | SL003761 | 1.09 | 0.76 (0.59-0.99) | 0.04 |
| C-C motif chemokine 8 | CCL8 | P80075 | SL000515 | -1.01 | 0.78 (0.62-0.99) | 0.04 |
| CD5 antigen-like | CD5L | O43866 | SL006108 | 1.30 | 1.23 (1.00-1.51) | 0.045 |
| Transforming growth factor-beta-induced protein ig-h3 | TGFBI | Q15582 | SL006544 | -1.00 | 0.82 (0.67-1.00) | 0.047 |
| Apolipoprotein A-I | APOA1 | P02647 | SL000019 | 1.01 | 0.80 (0.64-1.00) | 0.047 |
| Aromatic-L-amino-acid decarboxylase | DDC | P20711 | SL009216 | 1.14 | 1.28 (1.00-1.62) | 0.048 |

**Supplementary Table S2. Plasma proteins associated with endometriosis risk among endometriosis cases who had blood drawn within 2 years prior to their diagnosis and matched controls in the Nurses’ Health Study II (n=88)**

| **Protein** | **Entrez Gene Symbol** | **UniProt ID** | **SomaId** | **Fold change** | **Odds ratio (95%CI)** | **p-value** |
| --- | --- | --- | --- | --- | --- | --- |
| Neurexin-1-beta | NRXN1 | P58400 | SL009054 | -1.17 | 0.30 (0.14-0.65) | 0.002 |
| Neurexin-3-beta | NRXN3 | Q9HDB5 | SL008728 | -1.09 | 0.40 (0.22-0.75) | 0.004 |
| Interferon alpha/beta receptor 1 | IFNAR1 | P17181 | SL004475 | -1.12 | 0.07 (0.01-0.44) | 0.005 |
| Dipeptidyl peptidase 1 | CTSC | P53634 | SL007280 | -1.09 | 0.15 (0.04-0.56) | 0.005 |
| PILR alpha-associated neural protein | PIANP | Q8IYJ0 | SL019019 | -1.14 | 0.38 (0.19-0.75) | 0.005 |
| Neuronal growth regulator 1 | NEGR1 | Q7Z3B1 | SL008810 | -1.11 | 0.30 (0.13-0.70) | 0.005 |
| Netrin receptor UNC5D | UNC5D | Q6UXZ4 | SL005231 | -1.11 | 0.44 (0.25-0.79) | 0.006 |
| Histone H2B type 2-E | HIST2H2BE | Q16778 | SL014983 | 1.98 | 18.74 (2.28-153.86) | 0.006 |
| Desmocollin-2 | DSC2 | Q02487 | SL008631 | -1.09 | 0.38 (0.19-0.76) | 0.006 |
| Cadherin-6 | CDH6 | P55285 | SL004865 | -1.11 | 0.45 (0.25-0.80) | 0.006 |
| Histone H3.1 | HIST1H3A | P68431 | SL008158 | 2.08 | 2.70 (1.30-5.60) | 0.007 |
| Limbic system-associated membrane protein | LSAMP | Q13449 | SL005196 | -1.09 | 0.43 (0.23-0.80) | 0.008 |
| Melanoma-derived growth regulatory protein | MIA | Q16674 | SL001947 | -1.09 | 0.45 (0.25-0.83) | 0.01 |
| Dickkopf-related protein 3 | DKK3 | Q9UBP4 | SL009412 | -1.10 | 0.52 (0.31-0.86) | 0.01 |
| Hypoxia-inducible factor 1-alpha | HIF1A | Q16665 | SL003349 | -1.17 | 0.40 (0.20-0.81) | 0.01 |
| Cadherin-1 | CDH1 | P12830 | SL000055 | -1.06 | 0.42 (0.22-0.82) | 0.01 |
| Neuronal cell adhesion molecule | NRCAM | Q92823 | SL005210 | -1.12 | 0.42 (0.21-0.82) | 0.01 |
| Bcl-2-like protein 2 | BCL2L2 | Q92843 | SL003774 | -1.09 | 0.41 (0.21-0.82) | 0.01 |
| SLIT and NTRK-like protein 5 | SLITRK5 | O94991 | SL014070 | -1.13 | 0.32 (0.13-0.78) | 0.01 |
| Heat shock 70 kDa protein 1A | HSPA1A | P0DMV8 | SL000451 | 1.27 | 2.40 (1.21-4.78) | 0.01 |
| Dual specificity mitogen-activated protein kinase kinase 4 | MAP2K4 | P45985 | SL007237 | -1.08 | 0.49 (0.28-0.87) | 0.01 |
| Cerebral dopamine neurotrophic factor | CDNF | Q49AH0 | SL012538 | -1.11 | 0.00 (0.00-0.31) | 0.01 |
| WAP, Kazal, immunoglobulin, Kunitz and NTR domain-containing protein 2 | WFIKKN2 | Q8TEU8 | SL010391 | -1.09 | 0.44 (0.23-0.85) | 0.01 |
| Bone morphogenetic protein receptor type-1A | BMPR1A | P36894 | SL004080 | -1.09 | 0.47 (0.25-0.86) | 0.02 |
| Neural cell adhesion molecule 1, 120 kDa isoform | NCAM1 | P13591 | SL003764 | -1.08 | 0.50 (0.29-0.88) | 0.02 |
| Lactadherin | MFGE8 | Q08431 | SL006523 | -1.09 | 0.49 (0.27-0.87) | 0.02 |
| RGM domain family member B | RGMB | Q6NW40 | SL010468 | -1.07 | 0.42 (0.21-0.85) | 0.02 |
| Protein S100-A9 | S100A9 | P06702 | SL004477 | 1.41 | 2.42 (1.17-4.98) | 0.02 |
| RNA-binding protein 39 | RBM39 | Q14498 | SL011535 | 2.08 | 3.58 (1.26-10.22) | 0.02 |
| Cathepsin L2 | CTSV | O60911 | SL006910 | -1.13 | 0.35 (0.15-0.84) | 0.02 |
| Neural cell adhesion molecule L1 | L1CAM | P32004 | SL004154 | -1.10 | 0.41 (0.20-0.86) | 0.02 |
| Transforming growth factor beta receptor type 3 | TGFBR3 | Q03167 | SL005059 | -1.07 | 0.50 (0.28-0.90) | 0.02 |
| Low-density lipoprotein receptor-related protein 8 | LRP8 | Q14114 | SL004610 | -1.07 | 0.50 (0.28-0.90) | 0.02 |
| Urokinase-type plasminogen activator | PLAU | P00749 | SL000613 | -1.08 | 0.47 (0.24-0.90) | 0.02 |
| Insulin-like growth factor-binding protein 2 | IGFBP2 | P18065 | SL000466 | -1.04 | 0.48 (0.25-0.90) | 0.02 |
| Stromelysin-1 | MMP3 | P08254 | SL000524 | -1.09 | 0.46 (0.23-0.90) | 0.02 |
| Antithrombin-III | SERPINC1 | P01008 | SL000272 | -1.05 | 0.50 (0.28-0.91) | 0.02 |
| gp41 C34 peptide, HIV | Human-virus | Q70626 | SL016148 | -1.08 | 0.53 (0.30-0.92) | 0.02 |
| Sclerostin | SOST | Q9BQB4 | SL007631 | -1.07 | 0.48 (0.25-0.91) | 0.02 |
| Leucine-rich repeats and immunoglobulin-like domains protein 3 | LRIG3 | Q6UXM1 | SL010464 | -1.07 | 0.49 (0.26-0.91) | 0.02 |
| Interleukin-1 receptor type 2 | IL1R2 | P27930 | SL000145 | -1.06 | 0.50 (0.28-0.92) | 0.03 |
| Kallikrein-5 | KLK5 | Q9Y337 | SL003863 | -1.17 | 0.04 (0.00-0.68) | 0.03 |
| Myeloblastin | PRTN3 | P24158 | SL004008 | 1.62 | 2.95 (1.12-7.75) | 0.03 |
| Brother of CDO | BOC | Q9BWV1 | SL013490 | -1.07 | 0.47 (0.24-0.92) | 0.03 |
| Repulsive guidance molecule A | RGMA | Q96B86 | SL010467 | -1.05 | 0.55 (0.32-0.95) | 0.03 |
| Tumor necrosis factor receptor superfamily member 13B | TNFRSF13B | O14836 | SL004364 | 1.11 | 2.10 (1.06-4.14) | 0.03 |
| Transforming growth factor-beta-induced protein ig-h3 | TGFBI | Q15582 | SL006544 | -1.07 | 0.60 (0.38-0.96) | 0.03 |
| Carboxypeptidase E | CPE | P16870 | SL008703 | -1.14 | 0.02 (0.00-0.73) | 0.03 |
| Hepatoma-derived growth factor-related protein 2 | HDGFRP2 | Q7Z4V5 | SL014009 | 1.21 | 3.55 (1.10-11.45) | 0.03 |
| SPARC-related modular calcium-binding protein 1 | SMOC1 | Q9H4F8 | SL011888 | -1.04 | 0.53 (0.29-0.96) | 0.04 |
| Cytochrome c | CYCS | P99999 | SL000396 | -1.10 | 0.00 (0.00-0.59) | 0.04 |
| Leucine-rich repeat serine/threonine-protein kinase 2 | LRRK2 | Q5S007 | SL017128 | 1.08 | 1.89 (1.04-3.45) | 0.04 |
| Heterogeneous nuclear ribonucleoprotein Q | SYNCRIP | O60506 | SL007022 | 1.12 | 1.69 (1.02-2.82) | 0.04 |
| DNA repair protein RAD51 homolog 1 | RAD51 | Q06609 | SL006922 | -1.06 | 0.50 (0.26-0.98) | 0.04 |
| Interleukin-22 receptor subunit alpha-1 | IL22RA1 | Q8N6P7 | SL007806 | 1.27 | 2.97 (1.03-8.60) | 0.04 |
| Integrin alpha-I: beta-1 complex | ITGA1 ITGB1 | P56199, P05556 | SL003179 | 1.33 | 1.79 (1.01-3.17) | 0.05 |
| Delta-like protein 4 | DLL4 | Q9NR61 | SL010457 | -1.06 | 0.58 (0.34-0.99) | 0.05 |
| Mast/stem cell growth factor receptor Kit | KIT | P10721 | SL004010 | -1.06 | 0.58 (0.34-1.00) | 0.049 |

**Supplementary Table S3. Model fit statistics for multi-proteins models considering individual and combined proteins.**

| **Model** | **C-statistics (95%CI)** | **AIC** |
| --- | --- | --- |
| TOP1 | 0.65 (0.597, 0.704) | 264.583 |
| TOP1 IGFBP1 | 0.67 (0.617, 0.722) | 259.034 |
| S100A9 TOP1 | 0.664 (0.611, 0.716) | 260.141 |
| SERPINA3 | 0.624 (0.569, 0.678) | 267.642 |
| TOP1 NPPB | 0.652 (0.598, 0.705) | 262.849 |
| ANXA1 TOP1 | 0.674 (0.622, 0.726) | 261.103 |
| HIST1H3A TOP1 | 0.668 (0.615, 0.721) | 262.677 |
| RBM39 TOP1 | 0.662 (0.609, 0.715) | 263.514 |
| TOP1 GREM1 | 0.661 (0.608, 0.714) | 263.008 |
| S100A9 TOP1 IGFBP1 | 0.685 (0.634, 0.736) | 255.756 |
| RBM39 | 0.626 (0.572, 0.681) | 268.623 |
| RBM39 | 0.626 (0.572, 0.681) | 268.623 |
| S100A9 | 0.635 (0.581, 0.689) | 266.607 |
| S100A9 TOP1 CD5L | 0.685 (0.633, 0.736) | 257.896 |
| PRTN3 | 0.608 (0.552, 0.663) | 270.374 |
| ANXA1 TOP1 IGFBP1 | 0.695 (0.644, 0.746) | 256.723 |
| TOP1 IGFBP1 NPPB | 0.672 (0.62, 0.724) | 259.243 |
| MPO | 0.607 (0.551, 0.662) | 270.902 |
| HIST1H3A TOP1 IGFBP1 | 0.698 (0.647, 0.749) | 256.219 |
| S100A9 TOP1 NPPB | 0.668 (0.616, 0.721) | 260.466 |
| TOP1 GREM1 IGFBP1 | 0.696 (0.645, 0.747) | 257.259 |
| HIST1H3A | 0.643 (0.589, 0.697) | 269.034 |
| S100A9 TOP1 GREM1 | 0.687 (0.635, 0.738) | 258.474 |
| RBM39 TOP1 IGFBP1 | 0.684 (0.632, 0.735) | 258.024 |
| ANXA1 | 0.637 (0.583, 0.692) | 268.716 |
| S100A9 TOP1 IGFBP1 CD5L | 0.702 (0.652, 0.753) | 252.93 |
| PSMA2 | 0.608 (0.553, 0.663) | 270.952 |
| ANXA1 TOP1 GREM1 | 0.691 (0.639, 0.742) | 259.21 |
| ANXA1 TOP1 IGFBP1 CD5L | 0.705 (0.655, 0.755) | 254.204 |
| S100A12 | 0.6 (0.544, 0.655) | 272.695 |
| HIST1H3A TOP1 IGFBP1 CD5L | 0.713 (0.664, 0.763) | 252.162 |
| S100A9 GREM1 | 0.67 (0.618, 0.722) | 262.953 |
| RBM39 IGFBP1 | 0.652 (0.599, 0.706) | 263.326 |
| HIST1H3A IGFBP1 | 0.674 (0.621, 0.726) | 262.196 |
| RBM39 TOP1 IGFBP1 CD5L | 0.698 (0.648, 0.749) | 254.705 |
| S100A9 TOP1 GREM1 IGFBP1 | 0.712 (0.663, 0.762) | 253.986 |
| S100A9 TOP1 IGFBP1 NPPB | 0.682 (0.631, 0.734) | 257.146 |
| ANXA1 TOP1 GREM1 IGFBP1 | 0.718 (0.668, 0.767) | 254.757 |
| TOP1 GREM1 IGFBP1 NPPB | 0.697 (0.647, 0.748) | 257.429 |
| HIST1H3A TOP1 GREM1 IGFBP1 | 0.716 (0.666, 0.766) | 255.267 |
| S100A9 TOP1 GREM1 NPPB | 0.693 (0.642, 0.744) | 258.813 |
| S100A9 TOP1 IGFBP1 NPPB CD5L | 0.704 (0.653, 0.754) | 254.339 |
| S100A9 TOP1 GREM1 IGFBP1 CD5L | 0.725 (0.676, 0.774) | 251.736 |
| S100A9 HIST1H3A TOP1 IGFBP1 CD5L | 0.716 (0.667, 0.766) | 252.442 |
| HIST1H3A TOP1 IGFBP1 NPPB CD5L | 0.714 (0.664, 0.763) | 253.189 |
| ANXA1 TOP1 GREM1 IGFBP1 CD5L | 0.725 (0.676, 0.774) | 252.865 |
| HIST1H3A TOP1 GREM1 IGFBP1 CD5L | 0.729 (0.681, 0.778) | 251.846 |
| TOP1 GREM1 IGFBP1 NPPB CD5L | 0.713 (0.663, 0.762) | 254.954 |
| S100A9 TOP1 GREM1 IGFBP1 NPPB | 0.713 (0.663, 0.762) | 255.349 |
| ANXA1 TOP1 GREM1 IGFBP1 NPPB | 0.719 (0.67, 0.769) | 255.968 |
| HIST1H3A TOP1 GREM1 IGFBP1 NPPB | 0.717 (0.667, 0.766) | 256.063 |
| S100A9 TOP1 MFRP GREM1 IGFBP1 CD5L | 0.723 (0.674, 0.772) | 252.742 |
| S100A9 TOP1 GREM1 IGFBP1 NPPB CD5L | 0.728 (0.679, 0.777) | 253.085 |
| S100A9 TOP1 MPO GREM1 IGFBP1 CD5L | 0.726 (0.677, 0.775) | 253.046 |
| S100A9 HIST1H3A TOP1 IGFBP1 NPPB CD5L | 0.717 (0.667, 0.766) | 253.917 |
| S100A9 HIST1H3A TOP1 GREM1 IGFBP1 CD5L | 0.736 (0.688, 0.784) | 251.894 |
| ANXA1 TOP1 GREM1 IGFBP1 NPPB CD5L | 0.728 (0.679, 0.777) | 254.023 |
| ANXA1 TOP1 MPO GREM1 IGFBP1 CD5L | 0.724 (0.675, 0.773) | 253.85 |
| HIST1H3A TOP1 GREM1 IGFBP1 NPPB CD5L | 0.731 (0.682, 0.779) | 252.764 |
| S100A9 ANXA1 TOP1 GREM1 IGFBP1 CD5L | 0.73 (0.682, 0.779) | 253.292 |
| S100A9 TOP1 MFRP GREM1 IGFBP1 NPPB | 0.713 (0.663, 0.763) | 256.095 |
| S100A9 TOP1 MFRP GREM1 IGFBP1 NPPB CD5L | 0.726 (0.677, 0.775) | 253.949 |
| S100A9 TOP1 SERPINA3 GREM1 IGFBP1 NPPB CD5L | 0.728 (0.68, 0.777) | 254.768 |
| S100A9 TOP1 MPO GREM1 IGFBP1 NPPB CD5L | 0.727 (0.678, 0.776) | 254.632 |
| S100A9 TOP1 PRTN3 GREM1 IGFBP1 NPPB CD5L | 0.727 (0.678, 0.776) | 254.939 |
| S100A9 HIST1H3A TOP1 GREM1 IGFBP1 NPPB CD5L | 0.739 (0.691, 0.788) | 253.308 |
| S100A9 TOP1 GREM1 IGFBP1 NPPB CD5L HBA1HBB | 0.728 (0.679, 0.776) | 254.281 |
| HIST1H3A TOP1 MFRP GREM1 IGFBP1 NPPB CD5L | 0.731 (0.683, 0.78) | 253.78 |
| S100A9 TOP1 S100A12 GREM1 IGFBP1 NPPB CD5L | 0.733 (0.685, 0.781) | 253.559 |
| S100A9 ANXA1 TOP1 GREM1 IGFBP1 NPPB CD5L | 0.734 (0.685, 0.782) | 254.719 |
| ANXA1 TOP1 MPO GREM1 IGFBP1 NPPB CD5L | 0.725 (0.676, 0.774) | 255.355 |
| S100A9 TOP1 SERPINA3 S100A12 GREM1 IGFBP1 NPPB CD5L | 0.736 (0.688, 0.784) | 254.013 |
| S100A9 TOP1 SERPINA3 MFRP GREM1 IGFBP1 NPPB CD5L | 0.728 (0.679, 0.776) | 255.699 |
| S100A9 TOP1 MPO MFRP GREM1 IGFBP1 NPPB CD5L | 0.725 (0.676, 0.774) | 255.382 |
| S100A9 TOP1 MFRP S100A12 GREM1 IGFBP1 NPPB CD5L | 0.732 (0.684, 0.78) | 254.253 |
| S100A9 HIST1H3A TOP1 MFRP GREM1 IGFBP1 NPPB CD5L | 0.737 (0.689, 0.785) | 254.53 |
| S100A9 RBM39 TOP1 S100A12 GREM1 IGFBP1 NPPB CD5L | 0.748 (0.701, 0.795) | 252.237 |
| S100A9 HIST1H3A TOP1 S100A12 GREM1 IGFBP1 NPPB CD5L | 0.749 (0.702, 0.796) | 252.668 |
| S100A9 HIST1H3A TOP1 MPO GREM1 IGFBP1 NPPB CD5L | 0.736 (0.688, 0.784) | 254.977 |
| ANXA1 RBM39 TOP1 S100A12 GREM1 IGFBP1 NPPB CD5L | 0.744 (0.696, 0.792) | 254.184 |
| S100A9 HIST1H3A TOP1 GREM1 IGFBP1 NPPB CD5L HBA1HBB | 0.739 (0.691, 0.787) | 254.689 |
| S100A9 TOP1 SERPINA3 MFRP S100A12 GREM1 IGFBP1 NPPB CD5L | 0.736 (0.688, 0.783) | 254.834 |
| S100A9 RBM39 TOP1 MFRP S100A12 GREM1 IGFBP1 NPPB CD5L | 0.747 (0.699, 0.794) | 253.012 |
| S100A9 RBM39 TOP1 SERPINA3 S100A12 GREM1 IGFBP1 NPPB CD5L | 0.748 (0.701, 0.795) | 253.509 |
| S100A9 RBM39 TOP1 MPO S100A12 GREM1 IGFBP1 NPPB CD5L | 0.745 (0.698, 0.792) | 253.771 |
| S100A9 HIST1H3A TOP1 MFRP S100A12 GREM1 IGFBP1 NPPB CD5L | 0.747 (0.699, 0.794) | 253.854 |
| S100A9 HIST1H3A TOP1 MPO S100A12 GREM1 IGFBP1 NPPB CD5L | 0.746 (0.698, 0.793) | 254.113 |
| S100A9 RBM39 TOP1 S100A12 GREM1 IGFBP1 NPPB CD5L HBA1HBB | 0.756 (0.71, 0.803) | 252.64 |
| ANXA1 RBM39 TOP1 MFRP S100A12 GREM1 IGFBP1 NPPB CD5L | 0.746 (0.698, 0.793) | 254.886 |
| S100A9 HIST1H3A RBM39 TOP1 S100A12 GREM1 IGFBP1 NPPB CD5L | 0.756 (0.709, 0.802) | 252.841 |
| S100A9 ANXA1 RBM39 TOP1 S100A12 GREM1 IGFBP1 NPPB CD5L | 0.75 (0.703, 0.797) | 253.639 |
| S100A9 RBM39 TOP1 SERPINA3 MFRP S100A12 GREM1 IGFBP1 NPPB CD5L | 0.747 (0.7, 0.794) | 254.354 |
| S100A9 RBM39 TOP1 MPO MFRP S100A12 GREM1 IGFBP1 NPPB CD5L | 0.745 (0.697, 0.792) | 254.395 |
| S100A9 RBM39 TOP1 PRTN3 MFRP S100A12 GREM1 IGFBP1 NPPB CD5L | 0.745 (0.698, 0.792) | 254.637 |
| S100A9 HIST1H3A TOP1 MPO MFRP S100A12 GREM1 IGFBP1 NPPB CD5L | 0.744 (0.697, 0.792) | 255.163 |
| S100A9 RBM39 TOP1 MFRP S100A12 GREM1 IGFBP1 NPPB CD5L HBA1HBB | 0.754 (0.707, 0.8) | 254.041 |
| S100A9 HIST1H3A RBM39 TOP1 MFRP S100A12 GREM1 IGFBP1 NPPB CD5L | 0.755 (0.709, 0.802) | 253.952 |
| S100A9 ANXA1 RBM39 TOP1 MFRP S100A12 GREM1 IGFBP1 NPPB CD5L | 0.751 (0.704, 0.797) | 254.512 |
| S100A9 ANXA1 RBM39 TOP1 MPO S100A12 GREM1 IGFBP1 NPPB CD5L | 0.748 (0.701, 0.795) | 255.155 |
| S100A9 ANXA1 RBM39 TOP1 S100A12 GREM1 IGFBP1 NPPB CD5L HBA1HBB | 0.759 (0.712, 0.805) | 253.943 |
| S100A9 HIST1H3A RBM39 TOP1 S100A12 GREM1 IGFBP1 NPPB CD5L HBA1HBB | 0.761 (0.715, 0.807) | 253.609 |
| S100A9 RBM39 TOP1 MPO MFRP S100A12 GREM1 IGFBP1 NPPB CD5L HBA1HBB | 0.753 (0.706, 0.799) | 255.272 |
| S100A9 HIST1H3A RBM39 TOP1 MPO MFRP S100A12 GREM1 IGFBP1 NPPB CD5L | 0.752 (0.705, 0.799) | 255.392 |
| S100A9 HIST1H3A RBM39 TOP1 SERPINA3 MFRP S100A12 GREM1 IGFBP1 NPPB CD5L | 0.752 (0.706, 0.799) | 255.626 |
| S100A9 ANXA1 RBM39 TOP1 SERPINA3 MFRP S100A12 GREM1 IGFBP1 NPPB CD5L | 0.75 (0.703, 0.797) | 256.018 |
| S100A9 ANXA1 RBM39 TOP1 MPO MFRP S100A12 GREM1 IGFBP1 NPPB CD5L | 0.749 (0.702, 0.796) | 255.884 |
| S100A9 ANXA1 RBM39 TOP1 PRTN3 MFRP S100A12 GREM1 IGFBP1 NPPB CD5L | 0.748 (0.701, 0.795) | 256.235 |
| S100A9 ANXA1 RBM39 TOP1 MPO S100A12 GREM1 IGFBP1 NPPB CD5L HBA1HBB | 0.756 (0.709, 0.802) | 255.213 |
| S100A9 HIST1H3A RBM39 TOP1 MPO S100A12 GREM1 IGFBP1 NPPB CD5L HBA1HBB | 0.758 (0.712, 0.805) | 254.963 |
| S100A9 ANXA1 RBM39 TOP1 MFRP S100A12 GREM1 IGFBP1 NPPB CD5L HBA1HBB | 0.757 (0.711, 0.804) | 255.436 |
| S100A9 HIST1H3A RBM39 TOP1 MFRP S100A12 GREM1 IGFBP1 NPPB CD5L HBA1HBB | 0.76 (0.713, 0.806) | 255.168 |
| S100A9 ANXA1 RBM39 TOP1 SERPINA3 MPO MFRP S100A12 GREM1 IGFBP1 NPPB CD5L | 0.748 (0.701, 0.795) | 257.741 |
| S100A9 HIST1H3A RBM39 TOP1 MPO MFRP S100A12 GREM1 IGFBP1 NPPB CD5L HBA1HBB | 0.758 (0.712, 0.805) | 256.461 |
| S100A9 ANXA1 RBM39 TOP1 MPO MFRP S100A12 GREM1 IGFBP1 NPPB CD5L HBA1HBB | 0.756 (0.709, 0.803) | 256.64 |
| S100A9 ANXA1 RBM39 TOP1 MPO MFRP S100A12 GREM1 IGFBP1 NPPB HIST2H2BE CD5L | 0.752 (0.705, 0.799) | 257.478 |
| S100A9 ANXA1 RBM39 TOP1 SERPINA3 MFRP S100A12 GREM1 IGFBP1 NPPB CD5L HBA1HBB | 0.757 (0.71, 0.803) | 257.026 |
| S100A9 ANXA1 RBM39 TOP1 PRTN3 MPO MFRP S100A12 GREM1 IGFBP1 NPPB CD5L | 0.749 (0.702, 0.796) | 257.849 |
| S100A9 HIST1H3A RBM39 TOP1 PSMA2 MPO MFRP S100A12 GREM1 IGFBP1 NPPB CD5L | 0.751 (0.704, 0.798) | 257.071 |
| S100A9 ANXA1 HIST1H3A RBM39 TOP1 MPO MFRP S100A12 GREM1 IGFBP1 NPPB CD5L | 0.752 (0.705, 0.798) | 257.334 |
| S100A9 ANXA1 RBM39 TOP1 PSMA2 MPO MFRP S100A12 GREM1 IGFBP1 NPPB CD5L | 0.75 (0.703, 0.797) | 257.55 |
| S100A9 ANXA1 RBM39 TOP1 PRTN3 MFRP S100A12 GREM1 IGFBP1 NPPB CD5L HBA1HBB | 0.756 (0.709, 0.802) | 257.2 |
| S100A9 HIST1H3A RBM39 TOP1 SERPINA3 MPO MFRP S100A12 GREM1 IGFBP1 NPPB CD5L HBA1HBB | 0.758 (0.711, 0.804) | 258.424 |
| S100A9 ANXA1 RBM39 TOP1 SERPINA3 MPO MFRP S100A12 GREM1 IGFBP1 NPPB CD5L HBA1HBB | 0.755 (0.708, 0.802) | 258.574 |
| S100A9 ANXA1 RBM39 TOP1 MPO MFRP S100A12 GREM1 IGFBP1 NPPB HIST2H2BE CD5L HBA1HBB | 0.757 (0.711, 0.804) | 258.417 |
| S100A9 ANXA1 RBM39 TOP1 PSMA2 MPO MFRP S100A12 GREM1 IGFBP1 NPPB CD5L HBA1HBB | 0.758 (0.712, 0.804) | 258.003 |
| S100A9 HIST1H3A RBM39 TOP1 MPO MFRP S100A12 GREM1 IGFBP1 NPPB HIST2H2BE CD5L HBA1HBB | 0.759 (0.712, 0.805) | 258.452 |
| S100A9 ANXA1 RBM39 TOP1 PRTN3 MPO MFRP S100A12 GREM1 IGFBP1 NPPB CD5L HBA1HBB | 0.756 (0.709, 0.802) | 258.631 |
| S100A9 HIST1H3A RBM39 TOP1 PRTN3 MPO MFRP S100A12 GREM1 IGFBP1 NPPB CD5L HBA1HBB | 0.758 (0.712, 0.805) | 258.46 |
| S100A9 HIST1H3A RBM39 TOP1 PSMA2 MPO MFRP S100A12 GREM1 IGFBP1 NPPB CD5L HBA1HBB | 0.756 (0.71, 0.803) | 257.925 |
| S100A9 ANXA1 HIST1H3A RBM39 TOP1 MPO MFRP S100A12 GREM1 IGFBP1 NPPB CD5L HBA1HBB | 0.758 (0.712, 0.805) | 258.317 |
| S100A9 ANXA1 HIST1H3A RBM39 TOP1 PSMA2 MPO MFRP S100A12 GREM1 IGFBP1 NPPB CD5L | 0.751 (0.704, 0.798) | 258.956 |
| S100A9 ANXA1 RBM39 TOP1 SERPINA3 MPO MFRP S100A12 GREM1 IGFBP1 NPPB HIST2H2BE CD5L HBA1HBB | 0.758 (0.712, 0.805) | 260.39 |
| S100A9 ANXA1 RBM39 TOP1 SERPINA3 PSMA2 MPO MFRP S100A12 GREM1 IGFBP1 NPPB CD5L HBA1HBB | 0.758 (0.711, 0.804) | 259.953 |
| S100A9 ANXA1 RBM39 TOP1 PSMA2 MPO MFRP S100A12 GREM1 IGFBP1 NPPB HIST2H2BE CD5L HBA1HBB | 0.758 (0.712, 0.805) | 259.793 |
| S100A9 ANXA1 HIST1H3A RBM39 TOP1 SERPINA3 MPO MFRP S100A12 GREM1 IGFBP1 NPPB CD5L HBA1HBB | 0.758 (0.711, 0.804) | 260.293 |
| S100A9 ANXA1 RBM39 TOP1 PRTN3 MPO MFRP S100A12 GREM1 IGFBP1 NPPB HIST2H2BE CD5L HBA1HBB | 0.757 (0.711, 0.804) | 260.417 |
| S100A9 ANXA1 RBM39 TOP1 SERPINA3 PRTN3 MPO MFRP S100A12 GREM1 IGFBP1 NPPB CD5L HBA1HBB | 0.755 (0.709, 0.802) | 260.559 |
| S100A9 ANXA1 RBM39 TOP1 PSMA2 PRTN3 MPO MFRP S100A12 GREM1 IGFBP1 NPPB CD5L HBA1HBB | 0.758 (0.712, 0.804) | 260.003 |
| S100A9 ANXA1 HIST1H3A RBM39 TOP1 PSMA2 MPO MFRP S100A12 GREM1 IGFBP1 NPPB CD5L HBA1HBB | 0.759 (0.713, 0.806) | 259.648 |
| S100A9 ANXA1 HIST1H3A RBM39 TOP1 MPO MFRP S100A12 GREM1 IGFBP1 NPPB HIST2H2BE CD5L HBA1HBB | 0.759 (0.713, 0.806) | 260.279 |
| S100A9 ANXA1 HIST1H3A RBM39 TOP1 PRTN3 MPO MFRP S100A12 GREM1 IGFBP1 NPPB CD5L HBA1HBB | 0.758 (0.712, 0.805) | 260.317 |
| S100A9 ANXA1 RBM39 TOP1 SERPINA3 PSMA2 MPO MFRP S100A12 GREM1 IGFBP1 NPPB HIST2H2BE CD5L HBA1HBB | 0.758 (0.712, 0.805) | 261.773 |
| S100A9 ANXA1 HIST1H3A RBM39 TOP1 SERPINA3 PSMA2 MPO MFRP S100A12 GREM1 IGFBP1 NPPB CD5L HBA1HBB | 0.759 (0.713, 0.805) | 261.632 |
| S100A9 ANXA1 RBM39 TOP1 PSMA2 PRTN3 MPO MFRP S100A12 GREM1 IGFBP1 NPPB HIST2H2BE CD5L HBA1HBB | 0.759 (0.713, 0.805) | 261.783 |
| S100A9 ANXA1 RBM39 TOP1 SERPINA3 PRTN3 MPO MFRP S100A12 GREM1 IGFBP1 NPPB HIST2H2BE CD5L HBA1HBB | 0.758 (0.712, 0.805) | 262.361 |
| S100A9 ANXA1 HIST1H3A RBM39 TOP1 SERPINA3 MPO MFRP S100A12 GREM1 IGFBP1 NPPB HIST2H2BE CD5L HBA1HBB | 0.758 (0.712, 0.805) | 262.262 |
| S100A9 ANXA1 RBM39 TOP1 SERPINA3 PSMA2 PRTN3 MPO MFRP S100A12 GREM1 IGFBP1 NPPB CD5L HBA1HBB | 0.759 (0.713, 0.805) | 261.905 |
| S100A9 ANXA1 HIST1H3A RBM39 TOP1 PSMA2 MPO MFRP S100A12 GREM1 IGFBP1 NPPB HIST2H2BE CD5L HBA1HBB | 0.76 (0.714, 0.806) | 261.622 |
| S100A9 ANXA1 HIST1H3A RBM39 TOP1 SERPINA3 PRTN3 MPO MFRP S100A12 GREM1 IGFBP1 NPPB CD5L HBA1HBB | 0.758 (0.712, 0.805) | 262.271 |
| S100A9 ANXA1 HIST1H3A RBM39 TOP1 PSMA2 PRTN3 MPO MFRP S100A12 GREM1 IGFBP1 NPPB CD5L HBA1HBB | 0.759 (0.713, 0.806) | 261.637 |
| S100A9 ANXA1 HIST1H3A RBM39 TOP1 PRTN3 MPO MFRP S100A12 GREM1 IGFBP1 NPPB HIST2H2BE CD5L HBA1HBB | 0.76 (0.713, 0.806) | 262.278 |
| S100A9 ANXA1 RBM39 TOP1 SERPINA3 PSMA2 PRTN3 MPO MFRP S100A12 GREM1 IGFBP1 NPPB HIST2H2BE CD5L HBA1HBB | 0.759 (0.713, 0.805) | 263.7 |
| S100A9 ANXA1 HIST1H3A RBM39 TOP1 SERPINA3 PSMA2 MPO MFRP S100A12 GREM1 IGFBP1 NPPB HIST2H2BE CD5L HBA1HBB | 0.759 (0.713, 0.805) | 263.61 |
| S100A9 ANXA1 HIST1H3A RBM39 TOP1 SERPINA3 PSMA2 PRTN3 MPO MFRP S100A12 GREM1 IGFBP1 NPPB CD5L HBA1HBB | 0.76 (0.714, 0.806) | 263.567 |
| S100A9 HIST1H3A RBM39 TOP1 SERPINA3 PSMA2 PRTN3 MPO MFRP S100A12 GREM1 IGFBP1 NPPB HIST2H2BE CD5L HBA1HBB | 0.758 (0.712, 0.804) | 263.829 |
| S100A9 ANXA1 HIST1H3A RBM39 TOP1 PSMA2 PRTN3 MPO MFRP S100A12 GREM1 IGFBP1 NPPB HIST2H2BE CD5L HBA1HBB | 0.76 (0.714, 0.806) | 263.606 |
| S100A9 ANXA1 HIST1H3A RBM39 TOP1 SERPINA3 PRTN3 MPO MFRP S100A12 GREM1 IGFBP1 NPPB HIST2H2BE CD5L HBA1HBB | 0.759 (0.713, 0.806) | 264.234 |
| S100A9 ANXA1 HIST1H3A RBM39 TOP1 SERPINA3 PSMA2 PRTN3 MPO MFRP S100A12 GREM1 IGFBP1 NPPB HIST2H2BE CD5L | 0.753 (0.706, 0.8) | 264.795 |
| S100A9 ANXA1 HIST1H3A TOP1 SERPINA3 PSMA2 PRTN3 MPO MFRP S100A12 GREM1 IGFBP1 NPPB HIST2H2BE CD5L HBA1HBB | 0.749 (0.702, 0.796) | 265.945 |
| S100A9 ANXA1 HIST1H3A RBM39 TOP1 SERPINA3 PSMA2 PRTN3 MFRP S100A12 GREM1 IGFBP1 NPPB HIST2H2BE CD5L HBA1HBB | 0.762 (0.716, 0.808) | 263.991 |
| S100A9 ANXA1 HIST1H3A RBM39 TOP1 SERPINA3 PSMA2 PRTN3 MPO S100A12 GREM1 IGFBP1 NPPB HIST2H2BE CD5L HBA1HBB | 0.762 (0.716, 0.808) | 264.086 |
| S100A9 ANXA1 HIST1H3A RBM39 TOP1 SERPINA3 PSMA2 PRTN3 MPO MFRP S100A12 GREM1 IGFBP1 NPPB HIST2H2BE CD5L HBA1HBB | 0.76 (0.714, 0.806) | 265.538 |

Abbreviations: AIC, Akaike information criterion; CI, confidence interval

**Supplementary Table S4. Biological pathways associated with odds of endometriosis in the Nurses’ Health Study II**

| **Pathways** | **p-value** | **Activation z-score** | **Molecules included in the pathway** |
| --- | --- | --- | --- |
| Cell movement | 2.28E-16 | 2.428 | ALB,APOA1,CCL14,CCL16,CCL17,CCL8,CD5L,CD93,CDH1,CTSV,CXCL5,DKK3,DPT,HRG,HSPA1A/HSPA1B,ICAM2,IGF1,IGFBP1,IGFBP4,IL1R2,IL4,MAP2K3,MAP2K4,MB,NPPB,PRTN3,PTEN,RGMA,RSPO3,S100A9,SERPINC1,SPINT2,SYNCRIP,TFF2,TGFBI,TGFBR3,TNFRSF19,UNC5C,UNC5D,YWHAE |
| Migration of cells | 6.35E-16 | 2.584 | ALB,APOA1,CCL14,CCL16,CCL17,CCL8,CD5L,CD93,CDH1,CTSV,CXCL5,DPT,HRG,HSPA1A/HSPA1B,ICAM2,IGF1,IGFBP1,IGFBP4,IL1R2,IL4,MAP2K3,MAP2K4,NPPB,PRTN3,PTEN,RGMA,RSPO3,S100A9,SERPINC1,SPINT2,SYNCRIP,TFF2,TGFBI,TGFBR3,TNFRSF19,UNC5C,UNC5D,YWHAE |
| Leukocyte migration | 1.36E-13 | 2.314 | ALB,APOA1,CCL14,CCL16,CCL17,CCL8,CD5L,CD93,CDH1,CTSV,CXCL5,HRG,HSPA1A/HSPA1B,ICAM2,IL1R2,IL4,MAP2K3,NPPB,PRTN3,PTEN,RSPO3,S100A9,SERPINC1,SPINT2 |
| Cell movement of phagocytes | 1.83E-13 | 1.461 | ALB,APOA1,CCL14,CCL16,CCL17,CCL8,CDH1,CTSV,CXCL5,HRG,ICAM2,IL1R2,IL4,NPPB,PRTN3,PTEN,RSPO3,S100A9,SERPINC1,SPINT2 |
| Non-malignant disorder | 6.85E-12 | -0.232 | ALB,APOA1,CCL16,CDH1,CXCL5,DKK3,DPT,GDF11,GPC6,H3C1,HSPA1A/HSPA1B,ICAM2,IGF1,IGFBP1,IL1R2,IL4,MAP2K4,MB,NEGR1,NRXN3,PDE1A,PRTN3,PTEN,S100A9,SERPINC1,SPINT2,TGFBI,TGFBR3,UNC5D,WFIKKN2 |
| Cell movement of leukocytes | 1.27E-11 | 1.818 | ALB,APOA1,CCL14,CCL16,CCL17,CCL8,CD93,CDH1,CTSV,CXCL5,HRG,ICAM2,IL1R2,IL4,NPPB,PRTN3,PTEN,RSPO3,S100A9,SERPINC1,SPINT2 |
| Apoptosis | 2.06E-11 | -0.87 | ALB,APOA1,CD5L,CDH1,CTSV,DKK3,GDF11,HRG,HSPA1A/HSPA1B,ICAM2,IGF1,IGFBP1,IGFBP4,IL1R2,IL4,MAP2K3,MAP2K4,MB,NPPB,PI3,PIANP,PRTN3,PTEN,RGMA,RTN4R,S100A9,SERPINC1,SPINT2,SYNCRIP,TGFBI,TGFBR3,TNFRSF19,TOP1,UNC5C,YWHAE |
| Cell movement of myeloid cells | 2.35E-11 | 1.323 | ALB,APOA1,CCL14,CCL16,CCL17,CCL8,CTSV,CXCL5,ICAM2,IL1R2,IL4,NPPB,PRTN3,PTEN,RSPO3,S100A9,SERPINC1,SPINT2 |
| Rheumatoid arthritis | 3.41E-11 |  | ALB,APOA1,CCL14,CCL16,CCL17,CCL8,CDH1,CXCL5,GDF11,HSPA1A/HSPA1B,IGF1,IGFBP4,IL1R2,IL4,PRTN3,PTEN,S100A9,SERPINC1 |
| Cell movement of granulocytes | 3.66E-11 | 0.928 | ALB,APOA1,CCL14,CCL16,CCL17,CCL8,CXCL5,ICAM2,IL4,PRTN3,PTEN,RSPO3,S100A9,SERPINC1,SPINT2 |
| Non-traumatic arthropathy | 4.07E-10 |  | ALB,APOA1,CCL14,CCL16,CCL17,CCL8,CDH1,CXCL5,GDF11,HSPA1A/HSPA1B,IGF1,IGFBP4,IL1R2,IL4,LRIG3,PRTN3,PTEN,S100A9,SERPINC1 |
| Migration of phagocytes | 5.72E-10 | 0.284 | ALB,APOA1,CCL17,CCL8,CDH1,CTSV,CXCL5,ICAM2,IL4,PTEN,S100A9,SERPINC1 |
| Systemic autoimmune syndrome | 6.72E-10 | 1.067 | ALB,APOA1,CCL14,CCL16,CCL17,CCL8,CDH1,CTSV,CXCL5,DDC,DKK3,GDF11,HSPA1A/HSPA1B,IGF1,IGFBP4,IL1R2,IL4,PRTN3,PTEN,S100A9,SERPINC1,TGFBI |
| Inflammation of joint | 7.55E-10 | 0.057 | ALB,APOA1,CCL14,CCL16,CCL17,CCL8,CDH1,CXCL5,GDF11,HSPA1A/HSPA1B,IGF1,IGFBP1,IGFBP4,IL1R2,IL4,LRIG3,PRTN3,PTEN,S100A9,SERPINC1 |
| Immune mediated inflammatory disease | 1.05E-09 | 2.162 | ALB,APOA1,CCL14,CCL16,CCL17,CCL8,CDH1,CTSV,CXCL5,DKK3,GDF11,HSPA1A/HSPA1B,ICAM2,IGF1,IGFBP4,IL1R2,IL4,MAP2K3,PRTN3,PTEN,S100A9,SERPINC1,TGFBI |
| Amyloidosis | 1.08E-09 |  | ALB,APOA1,CTSV,DDC,HRG,HSPA1A/HSPA1B,IGF1,IL4,NRXN3,PDE1A,PTEN,RTN4R,S100A9,SERPINC1,TGFBI,TGFBR3 |
| Rheumatic Disease | 1.23E-09 | 0.057 | ALB,APOA1,CCL14,CCL16,CCL17,CCL8,CDH1,CXCL5,GDF11,HSPA1A/HSPA1B,ICAM2,IGF1,IGFBP1,IGFBP4,IL1R2,IL4,LRIG3,PRTN3,PTEN,S100A9,SERPINC1 |
| Migration of myeloid cells | 1.47E-09 | 0.824 | ALB,CCL16,CCL17,CTSV,CXCL5,ICAM2,IL4,PTEN,S100A9,SERPINC1 |
| Development of head | 1.79E-09 | 0.849 | CD93,CDH1,DPT,GDF11,HRG,IGF1,IGFBP1,IL4,LRIG3,MAP2K4,NEGR1,PTEN,RGMA,RTN4R,SMOC1,SPINT2,TGFBR3,UNC5C,WFIKKN2,YWHAE |
| Activation of phagocytes | 2.23E-09 | -0.384 | APOA1,CCL17,CCL8,CD93,CXCL5,IGF1,IL4,MAP2K3,PRTN3,PTEN,RGMA,S100A9,TFF2 |
| Endometriosis | 2.31E-09 |  | CCL16,CXCL5,DKK3,HSPA1A/HSPA1B,IGF1,IGFBP1,IL1R2,IL4,PDE1A,PTEN,S100A9,SPINT2,TGFBI |
| Activation of cells | 2.59E-09 | -0.399 | ALB,APOA1,CCL17,CCL8,CD93,CDH1,CXCL5,HRG,HSPA1A/HSPA1B,ICAM2,IGF1,IL4,MAP2K3,NPPB,PRTN3,PTEN,RGMA,S100A9,TFF2,TGFBR3 |
| Migration of granulocytes | 3.19E-09 | 0.551 | ALB,CCL16,CCL17,CXCL5,ICAM2,IL4,PTEN,S100A9,SERPINC1 |
| Chronic inflammatory disorder | 3.96E-09 |  | ALB,APOA1,CCL14,CCL16,CCL17,CCL8,CDH1,CXCL5,GDF11,HSPA1A/HSPA1B,IGF1,IGFBP4,IL1R2,IL4,NEGR1,PI3,PRTN3,PTEN,S100A9,SERPINC1 |
| Chemotaxis of myeloid cells | 5.14E-09 | 1.167 | APOA1,CCL14,CCL16,CCL17,CCL8,CXCL5,IL4,NPPB,PRTN3,PTEN,S100A9 |
| Chemotaxis of phagocytes | 5.62E-09 | 1.167 | APOA1,CCL14,CCL16,CCL17,CCL8,CXCL5,IL4,NPPB,PRTN3,PTEN,S100A9 |
| Cell movement of monocytes | 5.77E-09 | 1.584 | APOA1,CCL14,CCL16,CCL17,CCL8,CTSV,IL1R2,IL4,NPPB |
| Necrosis | 8.80E-09 | -1.01 | ALB,APOA1,CD5L,CDH1,CTSV,DKK3,HSPA1A/HSPA1B,IGF1,IGFBP1,IGFBP4,IL1R2,IL4,MAP2K3,MAP2K4,MB,NPPB,PI3,PIANP,PRTN3,PTEN,RBM39,RGMA,RTN4R,S100A9,SERPINC1,SYNCRIP,TGFBI,TGFBR3,TNFRSF19,TOP1,UNC5C,YWHAE |
| Chemotaxis | 9.75E-09 | 1.3 | APOA1,CCL14,CCL16,CCL17,CCL8,CXCL5,HRG,IGF1,IL4,NPPB,PRTN3,PTEN,S100A9,TFF2 |
| Development of vasculature | 9.77E-09 | 0.088 | APOA1,CDH1,CXCL5,HRG,IGF1,IGFBP1,IGFBP4,IL4,MAP2K3,MB,NPPB,PI3,PTEN,RGMA,RSPO3,S100A9,SERPINC1,TFF2,TGFBI,TGFBR3 |
